# Electronic Structure, Ligand Effects, and Chemical Reactivity of the Ground and Low-Lying Excited Electronic States of NpO^3+^

**DOI:** 10.3390/molecules31081258

**Published:** 2026-04-10

**Authors:** Taylor Gregory, Evangelos Miliordos

**Affiliations:** Department of Chemistry and Biochemistry, Auburn University, Auburn, AL 36849-5312, USA; tmg0076@auburn.edu

**Keywords:** neptunium oxide, ligand effects, methane oxidation, multi-reference calculations, density functional theory

## Abstract

Multi-reference and density functional theory calculations are performed for the diatomic and ligated NpO^3+^ species. The main goal of this study is to provide insights into the stability of the experimentally synthesized N(CH_2_CH_2_NR)_3_NpO (R = Si^i^Pr_3_) coordination complex and probe its use as a catalyst for the oxidation of methane. The constructed potential energy curves for NpO^3+^ showed the presence of three different types of minima (Np^3+^O, Np^4+^O^−^, Np^5+^O^2−^) depending on the neptunium–oxygen distance. All these minima are higher in energy than the Np^2+^ + O^+^ fragments, and the more stable Np^5+^O^2−^ form is stabilized only due to the presence of the negatively charged -CH_2_NR^−^ moiety of the ligand. The C–H bond activation of methane was found to be possible only for the first quintet state of the complex which lies about 30 kcal/mol higher than the ground triplet state.

## 1. Introduction

Transition metal coordination complexes have provided versatile machinery for achieving novel chemical transformations [1]. Optimization of the structures and functions of these complexes has created new avenues for the selective production and targeted design of inorganic and organic compounds that were previously impossible to make in high yields [1]. This progress enables us to see a future where current environmental, energy, and health-related challenges will be addressed by elegant chemical processes [1].

Transition metal oxides have been extensively probed for the selective conversion of methane to methanol [2]. The production of methanol from methane in high yields will enable the use of large quantities of natural gas (extracted at oil wells and fracking sites) as a feedstock, and not only as fuel [2]. Methanol is an industrially important solvent and can be used as the raw material for the synthesis of larger compounds used as commodity chemicals [2,3].

Despite intense research, selective viable catalysts have not been identified yet [2]. The main reason is that the C–H bond of the produced methanol is weaker than that of methane, and thus the employed catalysts over-oxidize methane to formaldehyde, formic acid, or even carbon dioxide. Research recently showed that kinetic (and not thermodynamic) factors may be able to revert this situation [4]. The prototypical (NH_3_)_4_RhO^2+^ coordination complex was found to provide a lower activation barrier for methane. This unusual reactivity was attributed to two factors: the electronic structure of the metal (Rh^4+^) center (low-spin high-oxidation state), which promotes the heterolytic dissociation of the activated C–H bond, and the formation of hydrogen bonds between the OH group of methanol and the NH groups of the ammonia ligands [4,5].

This work was motivated by the ongoing interest in the literature of identifying actinide complexes with high-oxidation states [6,7,8,9], and specifically the recently synthesized N(CH_2_CH_2_NR_2_)_3_NpO (R = Si^1^Pr_3_) coordination complex [10], which has a similar first coordination sphere and a NpO^3+^ functional unit with a formally Np^5+^ center. This higher oxidation state is expected to further favor the oxidation of methane. In addition, other interesting fundamental questions follow. Is the neptunium center indeed Np^5+^? What is the role of the *f*-electrons in the potential of the use of lanthanides and actinides as catalytic centers? How does the presence of *f*-electrons instead of d-electrons change the properties of the corresponding metal complexes? Besides the higher oxidation states that the *f*-block metal complexes can accommodate, they are expected to act differently from the *d*-block metal complexes for two more reasons: the larger range of coordination numbers for *f*-block metals and the stronger ionic character of the formed metal–ligand bonds [11]. Other *f*-block metal centers have been utilized for the activation of methane [12,13], but we are not aware of any studies probing *f*-block metal oxide complexes, while we are aware of only one more *f*-block metal-oxo complex [14].

Presently, we focus on the electronic structure of NpO^3+^ and that of the model compound N(CH_2_CH_2_NR)_3_NpO resembling the experimentally synthesized one. The model compound is computationally less demanding and is used to study its reaction with methane. The electronic structure of the neptunium center is probed with both multi-reference wavefunction- and density functional-based methodologies. First all atomic species from neutral Np to cationic Np^5+^ are studied, followed by the investigation of the NpO^3+^ diatomic cation, and, finally, model ligands are included to see their effects on the electronic structure and reactivity. Next, we describe the employed methodologies, followed by the presentation of our findings. Finally, we summarize our findings and provide insights for future studies.

## 2. Results and Discussion

### 2.1. Electronic Structure of NpO^3+^

The ground state of the neutral and the first five cationic neptunium species are Np (^6^L_11/2_; 5*f*^4^6*d*^1^7*s*^2^), Np^+^ (^7^L_5_; 5*f*^4^6*d*^1^7*s*^1^), Np^2+^ (^6^H_5/2_; 5*f*^5^), Np^3+^ (^5^I_4_; 5*f*^4^), Np^4+^ (^4^I_9/2_; 5*f*^3^), and Np^5+^ (^3^H_4_; 5*f*^2^). Going sequentially from Np to Np^5+^, our LC-SO-MRCI+Q-DKH3-calculated ionization energies compared to the experimental values in parentheses are 6.0 (6.266), 11.7 (11.5), 19.6 (19.7), 33.7 (33.8), and 49.2 (48.0) eV [15]. For the last two ionization energies, SC-SO-MRCI-DKH3 calculations were feasible, and the ionization energies changed by less than 0.1 eV (49.1 vs. 49.2 and 33.6 vs. 33.7 eV). The very good agreement between theory and experiment validates the methodology used presently. The calculated ionization energy for oxygen is 13.4 eV, which is also in very good agreement with the experimental value of 13.6 eV [15].

**Figure 1:**
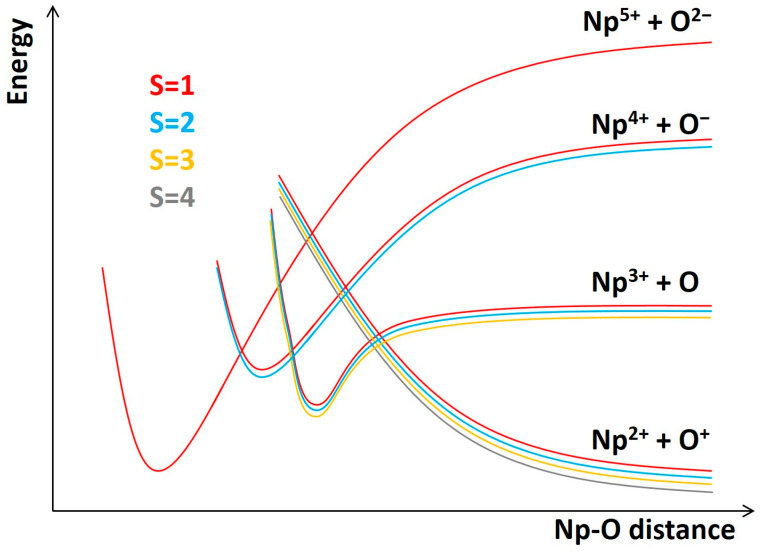
Quantitative plot of the expected potential energy curves coming from the ground states of the Np^2+^ + O^+^, Np^3+^ + O, Np^4+^ + O^−^, and Np^5+^ + O^2−^ channels. PECs of different spin multiplicities coming from the same channel are shifted for visibility.

Next, the potential energy curves (PECs), bonding schemes, electronic structure information, spin–orbit splitting, and spectroscopic constants for the lowest energy electronic states of the bare NpO^3+^ unit are discussed. Based on the ionization energies of neptunium and oxygen, and the electron affinity of oxygen, the lowest energy fragments are Np^2+^(^6^H) and O^+^(^4^S; *2p*^3^). This channel generates triplet, quintet, septet, and nonet states, ^3,5,7,9^[Σ^−^, Π, Δ, Φ, Γ, Η], all of which should have dissociative PECs due to the strong Coulombic repulsion. The next channel, Np^3+^(^5^I) + O(^3^P), produces triplets, quintets, and septets, which are expected to have moderately bound PECs. Two more channels of interest are Np^4+^(^4^I) + O^−^(^2^P) and Np^5+^(^3^H) + O^2−^(^1^S) since they are anticipated to form the equilibrium structures. A quantitative plot of the expected PECs for all four spin multiplicities is shown in Figure 1. Low-lying singlet states are omitted in this plot but they can be present stemming from the excited states of these channels.

**Figure 2:**
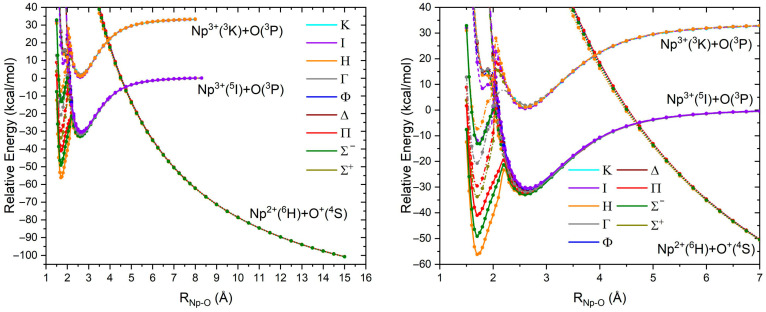
LC-MRCI-DKH3 PECs of NpO^3+^ as a function of the Np–O interatomic distance R_Np–O_. Curves depicted with short-dashed-dotted, solid, short-dash, dashed, and dotted lines correspond to singlet, triplet, quintet, septet, and nonet spin multiplicities, respectively. The top plot shows the PECs for a wider range of distances showing the Coulombically repulsive PECs of S = 4.

The constructed PECs at the LC-MRCI-DKH3 level shown in Figure 2 agree well with those of Figure 1. Thirty-four low-lying electronic states have been considered, i.e., ^1^Σ^+^, ^1^Π, ^1^Γ, ^1^Η, ^1^Ι, ^1^Κ, ^3^Σ^−^ (×2), ^3^Π, ^3^Δ, ^3^Φ, ^3^Γ, ^3^Η, ^3^Ι, ^5^Σ^−^, ^5^Π, ^5^Δ, ^5^Φ, ^5^Γ, ^5^Η, ^5^Ι, ^7^Σ^−^, ^7^Π, ^7^Δ, ^7^Φ, ^7^Γ, ^7^Η, ^7^Ι, ^9^Σ^−^, ^9^Π, ^9^Δ, ^9^Φ, ^9^Γ, and ^9^Η. The left figure includes distances from 1.0 to 15.0 Å, while the right figure offers a clearer view of the PECs at the equilibrium region. Individual plots for each spin multiplicity are given in Figure S1 in the Supporting Information (SI).

The PECs for the nonet spin multiplicity are indeed all repulsive following a 2/r asymptote. The same observation was found for the other spin states at the CASSCF level for long distances (6–7 to 15 Å), but in the region of 5–6 Å we coped with insurmountable technical issues due to the crossing of these repulsive PECs with the PECs coming from the next adiabatic channel, Np^3+^(^5^I; *5f*^4^) and O (^3^P; *2p*^4^). Since none of the Np^3+^ + O fragments can generate nonet states, the multi-reference character of nonet wavefunctions in the 5–6 Å is significantly suppressed easing the convergence issues. Therefore, for singlets, triplets, quintets, and septets, we used the wavefunction at 4 Å to construct the PECs. This strategy allowed us to get smooth PECs for distances between 1 and 9 Å. However, it should not be ignored that all these PECs are technically inaccurate in the 5–15 Å region, since they should suffer from an avoided crossing with the repulsive PECs of the Np^2+^(^6^H; 5*f*^5^) + O^+^(^4^S; *2p*^3^) channel at ~5 Å.

Focusing on Np^3+^(^5^I; *5f*^4^) + O (^3^P; *2p*^4^), we built the PECs of all produced states, ^3,5,7^[Σ^+^, Σ^−^(×2), Π(×3), Δ(×3), Φ(×3), Γ(×3), Η(×3), I(×2), K], at the CASSCF level of theory; see Figure S2 of SI. The PECs of all three spin multiplicities are all attractive, making potential energy wells with a minimum at around 2.7 Å. One third of these PECs are quasi-degenerate, making a beam of deeper potential wells with ~27 kcal/mol binding energy, while the other two-thirds of them create a second beam of PECs with ~10 kcal/mol binding energy. Looking at the CI vectors at the equilibrium distance of 2.7 Å, it is apparent that the most stable group of PECs corresponds to the 2*p_x_*^1^2*p_y_*^1^2*p_z_*^2^ configuration of O(^3^P), with z being the axis connecting the two atoms, while the other group pertains to 2*p_x_*^2^2*p_y_*^1^2*p_z_*^1^ or 2*p_x_*^1^2*p_y_*^2^2*p_z_*^1^. This justifies the relative ratio of states in the two groups. The lower energy group consists of the Σ^−^, Π, Δ, Φ, Γ, Η, Ι states, which can be formed from Np^3+^ (^5^I; M_L_ = 0, ±1, ±2, ±3, ±4, ±5, ±6) and O (^3^P; M_L_ = 0; 2*p_x_*^1^2*p_y_*^1^2*p_z_*^2^).

The PECs of the septet states are smooth along the whole range of distances, but the CASSCF PECs of the quintet states exhibit some inflection points at distances around 2 Å, and the PECs of triplets form a barrier at ~2.2 Å leading to new potential energy local minima at ~1.7 Å. At the MRCI, the observed inflection points become local minima (see for example the PEC of ^5^I in Figure 2), and the CASSCF local minima at 1.7 Å become global minima at the MRCI (compare Figure S3 of SI and Figure 2). The CI vectors of the quintet spin states at 2 Å reveal an ionic character where oxygen adopts a *2p*^5^ configuration, indicating that the observed inflection points are due to the involvement of the Np^4+^(^5^I; 5*f*^3^) + O^−^(^2^P; *2p*^5^) fragments, which can generate both quintets and triplets. Similarly, the minimum at 1.7 Å of the triplet states stems from Np^5+^(^3^H; 5*f*^2^) + O^2−^(^1^S; *2p*^6^), which can only produce triplet states. The latter channel generates the ^3^[Σ^−^, Π, Δ, Φ, Γ, Η] states, with ^3^H, ^3^Σ^−^, and ^3^Π creating the lowest energy states of NpO^3+^.

The next lowest energy minima correspond to the PECs of singlet spin states, ^1^Σ^+^, ^1^Π, ^1^Γ, ^1^Η, and ^1^I (see Figure 2). The PECs stem from the next asymptotic channel corresponding to the first triplet state of Np^3+^(^3^K) and O(^3^P). To our knowledge, there is no experimental report on an excited state of Np^3+^; the calculated Np^3+^(^3^K) state has a 5*f*^4^ character. The PECs of the singlet states are nearly parallel to the triplet states with the same features, such as local minima at 2.7 Å, avoided crossings at ~2 Å, and lower energy minima at ~1.7 Å. Based on the CI vectors, the latter minima also originate from Np^5+^ + O^2−^.

**Figure 3:**
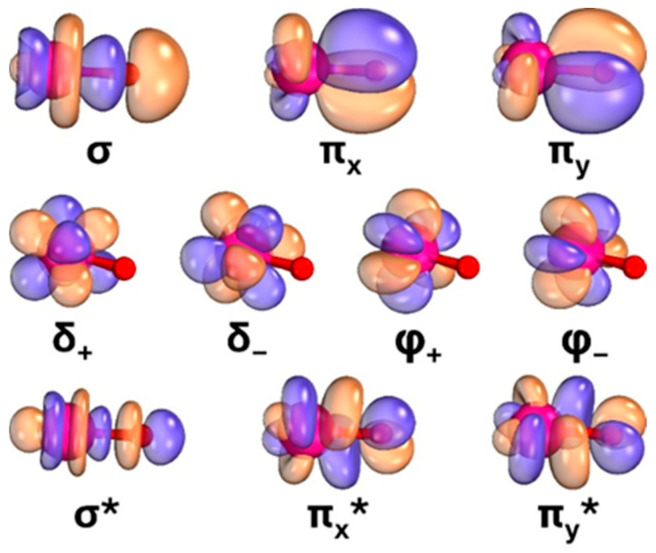
Contours of the molecular orbitals of NpO^3+^ at the equilibrium distance of 1.7 Å.

The main configurations of the CI vector of the lowest triplet and singlet spin states at 1.7 Å are listed in Table 1, and the corresponding molecular orbitals are shown in Figure 3. The σ and π_x,y_ orbitals pertain to the bonding Np–O orbitals. The compositions of these are σ ≈ 0.81 [2*p_z_*(O)] − 0.51 [4*f_z_*^3^(Np)] and π_x,y_ ≈ 0.81 [2*p_x,y_*(O)] + 0.35 [4*f_xz_*^2^,*_yz_*^2^(Np)]. The σ* and π_x,y_* are the corresponding anti-bonding orbitals, σ* ≈ 0.50 [2*p_z_*(O)] + 0.81 [4*f_z_*^3^(Np)] and π_x,y_* ≈ 0.81 [2*p_x,y_*(O)] − 0.73 [4*f_xz_*^2^,*_yz_*^2^(Np)]. The bonding orbitals are polarized more towards the oxygen terminus, while the antibonding ones are polarized towards neptunium. The remaining four orbitals, δ_±_ and φ_±_, are non-bonding atomic orbitals of neptunium since they have minimal overlap with the valence orbitals of oxygen. This pattern resembles that of transition metal oxide dications [16], with the difference that φ_±_ are present only for *f*-block metals.

In all nine states listed in Table 1, the σ and π orbitals are doubly occupied (natural orbital population is larger than 1.92) and the corresponding σ* and π* are vacant (natural orbital population is smaller than 0.08). This observation signifies that the oxygen terminus for all these states has a strong O^2−^ character (σ and π are heavily localized on oxygen). The remaining two electrons populate the δ_±_ and φ_±_ orbitals, and the specific combinations determine the overall symmetry and angular momentum of the wavefunctions. For example, the wavefunctions of the ^1,3^H and ^1,3^Π states are composed of the various δ_+_^1^φ_+_^1^/δ_−_^1^φ_−_^1^ configurations (B_1_ symmetry component), whereas the ^1^Γ, ^1^I, ^1^Σ^+^, and ^3^Σ^−^ include δ_+_^1^δ_−_^1^/φ_+_^1^φ_−_^1^ electron configurations (see Table 1).

To further understand the bonding scheme of the potential wells at 2.7 Å, we plotted the orbital contours in Figure S3 of SI. At this distance, there is minimal overlap between the atomic orbitals of neptunium and oxygen. The σ and π orbitals are clearly localized on oxygen and are practically identical to its 2*p* orbitals. The remaining orbitals are all localized on neptunium and have mainly 5*f* character. The wavefunctions for all spin multiplicities are very multi-reference and the CI vectors include exclusively σ^2^π^2^ or σ^1^π^3^ (S = 1) configurations for oxygen corresponding to O (^3^P; 2*p*^4^). As a result, the natural populations for σ and π or *2p* are 4/3 = 1.333, and for the other orbitals (σ*, π*, δ, φ or 5f) are 4/7 = 0.571. These observations point to Np^3+^–O electrostatically attracted structures.

For distances of ~1.9 Å, the quintet states reveal some shallow minima or inflection points with CI vectors composed mainly of σ^2^π^3^ or σ^1^π^4^ (S = 1/2) configurations pertaining to Np^4+^O^−^ species. Triplet states can also be generated from Np^4+^(^4^I; 5*f*^3^) + O^−^(^2^P), but the corresponding minima are shadowed by the crossings with the Np^5+^(^3^H; 5*f*^2^) + O^2−^(^2^P) PECs. There are no quintet states generated by the latter channel, rendering the Np^4+^O^−^ minima particularly clear for quintets. There are no septet states from either Np^4+^O^−^ or Np^5+^O^2−^ and thus only Np^3+^O minima are observed for septets. Overall, the constructed PECs for NpO^3+^ reveal the existence of four different electronic structure patterns depending on the spin multiplicity and Np–O distance. For distances longer than 5.0 Å the dissociative Np^2+^O^+^ dominates for all spin states (S = 0, 1, 2, 3, 4); for distances of ~2.7 Å there are Np^3+^O minima for S = 0, 1, 2, 3, for distances of ~1.9 Å there are shallow Np^4+^O^−^ minima for S = 2, and for distances of ~1.7 Å the S = 0 and 1 states are dominant.

Focusing on the Np^5+^O^2−^ minima, SO calculations are performed for Np–O distances between 1.6 and 1.85 Å. The PECs are shown in Figure S4 of SI, while equilibrium distances, harmonic vibrational frequencies, excitation energies, and analysis of the wavefunction in terms of the parent ^2S+1^Λ states are listed in Table 2. The lowest energy state can be fairly written as a 1^3^H_4_ state as it has Ω = 4 and it is composed of 1^3^Η by 97%. The next two states at 0.383 and 0.633 eV have Ω = 0^+^ and 1 and cannot be assigned to a specific ^2S+1^Λ state. But the next two states at 0.878 and 1.298 eV can be clearly represented as 1^3^H_5_ and 1^3^Π_0−_. Eleven more states are listed in Table 2 with excitation energies ranging from 1.391 to 3.035 eV, and only five of them remain rather pure ^2S+1^Λ states: 1^3^H_6_, 1^3^Π_2_, 1^1^Γ_4_, 1^1^Ι_6_, and 1^1^Η_5_. The equilibrium bond lengths r_e_ of all these states are bracketed between 1.709 and 1.743 Å, and the frequencies ω_e_ cover the range of 810–878 cm^−1^. The similar r_e_ and ω_e_ values mirror the same bonding character in all states (Np^5+^O^2−^ with minor influence from the non-bonding *f*-electrons of neptunium) and indicate nearly parallel PECs with large Franck–Condon factors. To see the effect of correlating the subvalence 6s and 6p electrons, we performed calculations without correlating them for the ground state ^3^H. The bond lengths increased by 0.009 Å.

### 2.2. Ligand Effects on the Stabilization of NpO^3+^

Figure 2 indicates that the equilibrium Np^5+^O^2−^ structure is metastable lying higher than the lowest energy fragments Np^2+^ + O^+^. To understand the role of the ligands in stabilizing the experimentally observed N(CH_2_CH_2_NR)_3_NpO, R = Si^i^Pr, we performed calculations on the model (NH_3_)_x_(NH_2_^−^)_y_Np^5+^O^2−^ and (NH_3_)_x_(NH_2_^−^)_y_Np^2+^ species with x = 0–1 and y = 0–3. For all species different spin multiplicities (see Table S1 of SI) were considered. The geometry of the model systems was made starting with N(CH_2_CH_2_NR)_3_NpO, keeping only the nitrogen and oxygen atoms, and saturating the N atoms with hydrogen atoms. Here we focus on the lowest energy spin state, which is found to be S = 1 for all oxides, and S = 5/2 for the ligated Np^2+^ species except for x = 0/y = 3, which favors the S = 3/2 state.

**Figure 4:**
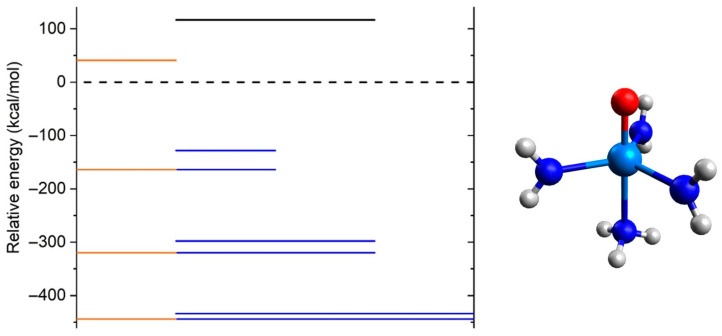
B3LYP relative energy of the model (NH_3_)_x_(NH_2_^−^)_y_Np^5+^O^2−^ species (x = 0–1, y = 0–3) with respect to (NH_3_)_x_(NH_2_^−^)_y_Np^3+^ + O^+^ (dashed line). For convenience the length of the red/blue lines represents the x/y values. The case of x = 0 and y = 0 is shown with a black solid line.

The energy difference at B3LYP/RSC(Np)/cc-pVTZ(N,H)/aug-cc-pVTZ(O) between (NH_3_)_x_(NH_2_^−^)_y_Np^5+^O^2−^ and (NH_3_)_x_(NH_2_^−^)_y_Np^2+^ + O^+^ is plotted in Figure 4. For NpO^3+^ (x = 0/y = 0) the equilibrium structure is unstable which agrees with the PECs of Figure 2. The addition of the axial NH_3_ stabilizes the equilibrium but remains unstable. However, the addition of one equatorial NH_2_^−^ makes it stable by 128.1 kcal/mol; this trend continues with additional equatorial NH_2_^−^ ligands. The second NH_2_^−^ stabilizes it further by 169.1 kcal/mol and a third one by 135.9 kcal/mol more. As a result, the latter species is overall stabilized by an energy of 433.9 kcal/mol. The addition of the ammonia ligand stabilizes (NH_2_^−^)_y = 1–3_Np^5+^O^2−^ by no more than 36.1 kcal/mol (y = 1). The stabilization energy due to ammonia decreases as y increases and is only 10.4 kcal/mol for y = 3 (see Figure 4). In conclusion, the NH_3_ ligand has a minimal effect on the overall stability of the complex but serves as an anchor for the polydentate ligand and the NH_2_^−^ units, which are responsible for stabilizing the Np^5+^O^2−^ unit. Similar observations have been made for Fe^4+^O^2−^ vs. Fe^3+^O^−^ units, where ammonia ligands were shown to stabilize Fe^4+^O^2−^ [17]. These examples show how ligand design can be wisely selected to stabilize unbound structures with high-oxidation state metal centers. For reasons of completeness, the binding energy of (NH_3_)(NH_2_^−^)_3_Np^5+^O^2−^ with respect to the lowest energy fragments, (NH_3_)_x_(NH_2_^−^)_y_Np^3+^(S = 2) + O(^3^P), is calculated to be 127.8 kcal/mol, which is indicative of a strong metal–oxygen bond [18,19].

**Figure 5:**
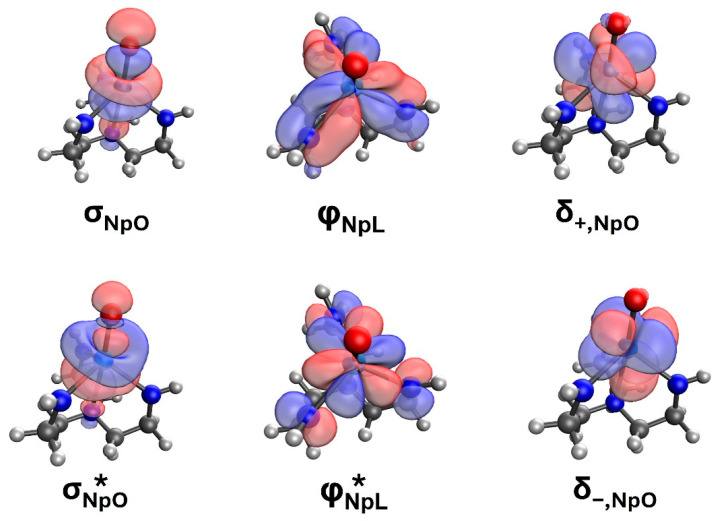
Contours of the partially occupied natural orbitals of N(CH_2_CH_2_NH)_3_NpO.

Finally, the electronic structure of the fully coordinated complex is examined. The six partially occupied natural orbitals (DFT/B3LYP level) of the fully coordinated complex are shown in Figure 5. These orbitals can be divided into three groups: $\sigma_{NpO}$ and $\sigma_{NpO}^{\;*}$, *δ_+,NpO_* and *δ_−,NpO_*, and $\varphi_{NpL}$ and $\varphi_{NpL}^{\;*}$. The first group pertains to the bonding and anti-bonding orbitals of the NpO σ-bond, which are populated by 1.985 and 0.015 electrons. The second group includes the non-bonding δ-orbitals like those observed for pure NpO^3+^, and they are singly occupied. The third group combines the *f*_φ-_orbitals of pure NpO^3+^ with the p_π_ of the three −CH_2_NH− groups forming in-phase and out-of-phase combinations representing donation and back-donation schemes [20]. Their natural occupations are also 1.975 (in-phase) and 0.025 (out-of-phase) electrons. A population of $\sigma_{NpO}^{\;1.9\;}\varphi_{NpL}^{\;1.9\;}\delta_{+,NpO}^{\;1.0\;}\delta_{-,NpO}^{\;1.0\;}$ points to a “pure” Np^5+^O^2−^ unit, specifically the 1^3^Σ^−^ state of NpO^3+^ (see Table 1), and three −CH_2_NH^−^ units donating electrons to the vacant $\varphi_{+,NpO}$ orbital. The overall bonding scheme suggests minor (−CH_2_NH)_3_^2−,●^Np^4+^O^2−^ character, which would be important if the population of $\varphi_{NpL}^{\;*}$ were larger.

Next, higher-level multi-reference state-averaged (including six triplet states) CASSCF and MRCI calculations for its lowest energy electronic states were carried out to further shed light on the electronic structure of N(CH_2_CH_2_NH)_3_NpO. Several attempts to include the $\sigma_{NpO}$, $\sigma_{NpO}^{\;*}$, $\varphi_{NpL}$, and π_NpO_ orbitals in the CASSCF active space failed since these orbitals remained as closed-shell orbitals confirming the strong (−CH_2_NH^−^)_3_Np^5+^O^2−^ nature of the complex. Our final CASSCF active space is composed of two electrons in seven orbitals corresponding to the δ_±_, φ_±_, π_x,y_*, and σ* orbitals of Figure 3 (*φ_+_ ~*$\varphi_{NpL}^{*\;}$). In the subsequent MRCI calculations 22 electrons are correlated pertaining to the two aforementioned electrons and all seven lone pairs of nitrogen atoms.

According to the MRCI results, the lowest energy states are triplet states, well separated from singlet or quintet states. The CI vectors (CI coefficients in parentheses) indicate that the ground state is of mixed character [δ_−_^1^φ_+_^1^ (0.70), δ_+_^1^δ_−_^1^ (0.41), δ_+_^1^φ_−_^1^ (−0.44)] followed by another two multi-reference states, [δ_+_^1^φ_+_^1^ (0.79), δ_−_^1^φ_−_^1^ (0.54)] and [δ_+_^1^δ_−_^1^ (0.75), δ_−_^1^φ_+_^1^ (−0.41)], at 0.002 and 0.047 eV (MRCI+Q) excitation energies. All these electron configurations are present in the first three states of NpO^3+^ (1^3^H, 1^3^Σ^−^, 1^3^Π), while the DFT/B3LYP natural orbital populations fail to mirror the multi-reference character of these states. Our results are somewhat different from the CASSCF/CASPT2 calculations reported by Dutkiewicz et al. [5], who found a ground state with a configuration described as φ_+_^1^φ_−_^1^ in the present notation (see Figure S29 of their SI). Their DFT electron configuration (~δ_+_^1^δ_−_^1^; Figure S30 of their SI) agrees with the present DFT results. Finally spin–orbit calculations were conducted combining the MRCI spin–orbit Hamiltonian elements with MRCI+Q energies. The spin–orbit operator further mixed these three states splitting them into eighteen states lying in an energy range of 1.66 eV (see Table S2 of SI).

### 2.3. Assessment of Neptunium Oxide as Catalyst for Methane Activation

Recently, our group identified the (NH_3_)_4_Rh^4+^O^2−^ trigonal bipyramidal as a potential catalyst for selectively converting methane into methanol [3,4]. The main feature of this catalyst is that it promotes the [2+2] mechanism instead of the radical mechanism and that further oxidation of the produced methanol is prevented kinetically. The observed trend was attributed to the low-spin high-oxidation state Rh^4+^ center. The present N(CH_2_CH_2_NH)_3_NpO molecular complex presents similar geometric and electronic structure (trigonal bipyramidal and high-oxidation state of the metal). Therefore, its ability to activate methane was assessed for the two mechanisms ([2+2] and radical) and for the different spin states. The DFT calculations (see Section 3) used for this purpose should be considered as qualitative since the multi-reference character even for the ground state (see Section 2.2) and spin–orbit effects should be considered for more accurate calculations.

**Figure 6:**
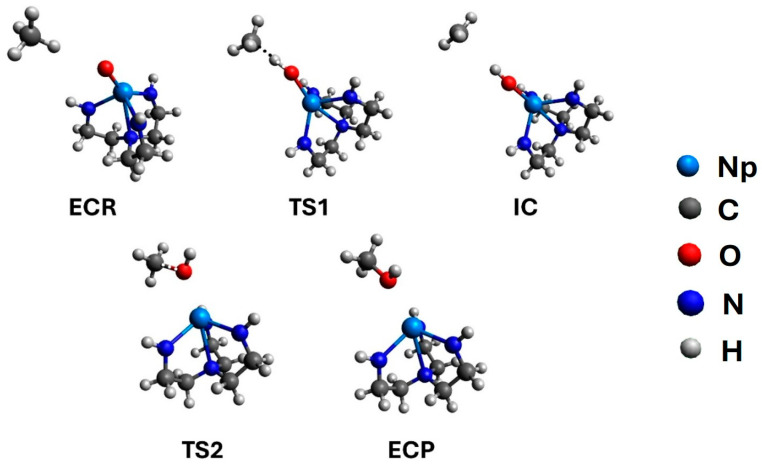
B3LYP/RSC structures of all intermediate (ECR, IC, ECP) and transition states (TS1, TS2) of the N(CH_2_CH_2_NH)_3_NpO (S = 1) + CH_4_ reaction (radical mechanism).

The structures of the radical mechanism (S = 1) for the initial encounter complex of the reactants (ECR), the intermediate complex (IC), the transition state (TS1) connecting them, the encounter complex of the products (ECP) and the transition state (TS2), which connects the IC and ECP, are shown in Figure 6. The pathway from the IC to TS2 involves the rotation of the OH group. The Np–O-H angle of 180° changes to about 110° allowing the CH_3_ group to approach oxygen. We were not able to optimize all the corresponding structures for the [2+2] mechanism. The Cartesian coordinates for the ECR, TS1, and IC are given in Tables S3 and S4 of the SI (for all spin multiplicities, S = 0, 1, 2, 3) along with all structures of the radical path. However, all our attempts to optimize the TS2 structure for [2+2] led to the TS2 structure of the radical mechanism.

The complete energy diagrams along the reaction coordinate at B3LYP, MN15, MN15 combined with solvent (water or toluene) effects are given in the SI (Figures S5–S8). MN15 seems to stabilize the IC, ECP, products of the low spin states (S = 0,1) and the TS1 (S = 1) structure of [2+2] over the radical pathway. The solvent effects using a polar (water) or non-polar (toluene) primarily favor the release of methanol, reducing the binding energy of methanol to the metal center.

The gas-phase MN15-calculated H_3_C–H activation barriers (ECR→TS1) are relatively large (>35 kcal/mol) for the lowest energy spin states (S = 0, 1) and both reaction paths ([2+2] or radical). The next higher spin state (S = 2) bears small H_3_C–H activation and H3C–OH (IC→TS2) recombination barriers of 16.3 and 22.8 kcal/mol (radical mechanism), respectively, and its potential energy surface crosses with those of S = 0 and S = 1. The first S = 3 also has small activation barriers but lies higher in energy and is well separated from the others. Among the S = 0, 1, 2 states, the quintet is the only state where the ECP is lower in energy than the ECR.

According to the singly occupied orbitals of the ECR and IC for S = 2 (see Figure S9 of SI), the distinct reactivity of S = 2 can be attributed to the radical character on the amidic terminals (-NH) of the polydentate ligand (see Figure S6 of the SI). The -NH^●^ radical accepts readily an electron from methane to return to -NH^−^, while the terminal oxygen accepts a proton from methane (see Figure S9 of the SI).

This proton-coupled electron transfer process is like the one observed in transition metal oxides [2,11], but the new feature in the present complex is that this electron transfer is mediated by ligand species.

The NpO and Np–N bond lengths are indicative of the electronic structure of N(CH_2_CH_2_NH)_3_NpO for S = 0, 1, 2, 3. The Np–O bond distances for S = 0–3 are 1.82, 1.82, 1.86, and 2.27 Å, and the Np–N_ax_ distances of the quaternary axial nitrogen are 2.67, 2.68, 2.76, and 2.76 Å. The other Np–N_eq_ (equatorial nitrogen atoms) distances range within 2.21–2.23 Å for both S = 0 and S = 1. The S = 2 state has one long Np–N bond (2.41 Å) and two shorter bonds of 2.27 Å. The S = 3 state has two long and one short Np–N bonds of 2.41–2.42 Å and 2.27 Å, respectively. The corresponding values for the N(CH_2_CH_2_NSiH_3_)_3_NpO (S = 1) complex taken from ref. [5] are 1.89 (Np–O), 2.67 (Np–N_ax_), and 2.25 (Np–N_eq_) Å. The latter Np–N_ax_ and Np–N_eq_ values are in very good agreement with the present ones, but the Np–O bond is more elongated than the present one, by 0.07 Å, probably due to the presence of the silicon groups.

**Figure 7:**
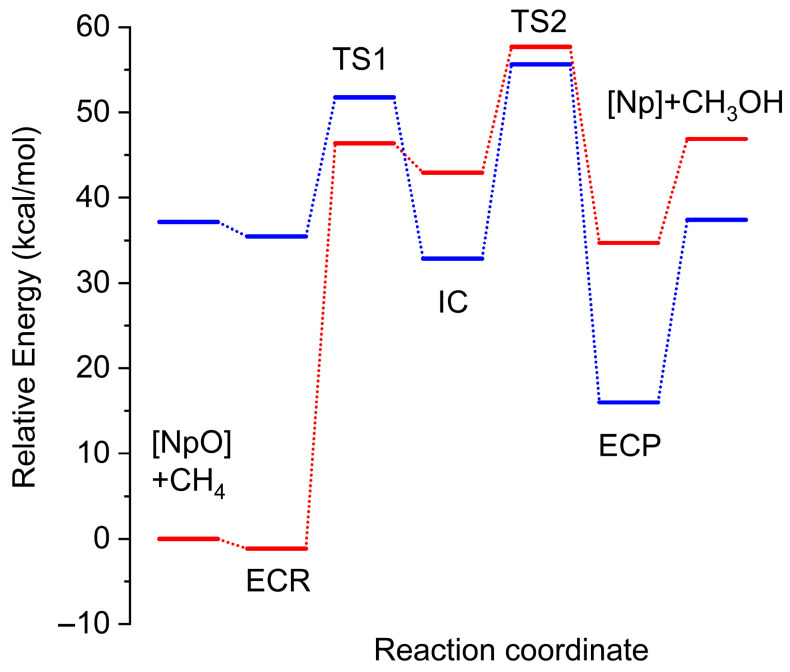
MN15/RSC//B3LYP/RSC energy diagram for the radical mechanism of the N(CH_2_CH_2_NH)_3_NpO (S = 1 in red and S = 2 in blue) + CH_4_ reaction (see Figure 6) for the S = 1 structures of the ECR, TS1, IC, TS2, and ECP.

The potential energy profiles for S = 1 and S = 2 plotted in Figure 7 cross at the geometry of the IC due to the very similar electronic structure, where two neptunium electrons couple with the single remote electron of methyl, N(CH_2_CH_2_NH)_3_NpOH (S = 3/2) × CH_3_ (S = 1/2). Overall, the lowest energy ECR (S = 1) is 17 kcal/mol lower in energy than the lowest energy ECP (S = 2) preventing the use of N(CH_2_CH_2_NH)_3_NpO as a catalyst for methane activation. This highly endothermic process can be attributed to the strong NpO triple (σ^2^π^4^) bond (D_e_ = 127.8 kcal/mol; see Section 2.2), which is reluctant to provide the oxygen atom to oxidize methane. Therefore, a suggested strategy for utilizing *f*-block metals in methane activation would be the use of proper ligands that host an unpaired electron to stabilize a high-spin state and render it a ground state.

## 3. Computational Methods

The calculations on the atomic species Np though Np^5+^ were performed at the multi-reference configuration interaction (MRCI) level of theory. The active space of the reference complete active space self-consistent field (CASSCF) wavefunction consisted of the 5*f*, 6*d*, 7*s* orbitals. At the MRCI level, two sets of calculations were carried out, where, in addition to the valence electrons, the 5*s*, 5*p*, 5*d*, 6*s*, 6*p* electrons (small core = SC) or the 6*s*, 6*p* electrons (large core = LC) are also correlated. To account for scalar relativistic effects, the third order Douglas–Kroll–Hess (DKH3) transformation was employed [21]. The spin–orbit (SO) relativistic effects were considered by diagonalizing the Breit–Pauli Hamiltonian in the basis of the DHK3-relativistic MRCI wavefunctions [22]. To improve the accuracy of the calculations, the MRCI energies were replaced with the MRCI+Q energies in the diagonal elements. Due to the computational requirements, we were unable to obtain spin–orbit results with SC for Np^2+^, Np^+^, and Np. For these calculations, the all-electron cc-pwCVTZ-DK3 basis set was used [23], and the internally contracted MRCI scheme was used as implemented in MOLPRO 2021.3 [24].

MRCI calculations were also carried out for the NpO^3+^ cation with MOLPRO. Since only the 5*f* orbitals are populated in the higher oxidation states of neptunium, the CASSCF active space included these 5*f* orbitals and the 2*p* orbitals of oxygen. The C_2v_ point group was exploited, and the active space corresponds to three a_1_, three b_1_, three b_2_, and one a_2_ orbitals. To achieve properly converged wavefunctions, we started with calculations for the triplet spin states at 4.0 Å and conducted the necessary orbital rotations. As explained above, the distance of 4.0 Å avoids crossing between states from different asymptotic channels offering easier convergence. Then potential energy curves were constructed along the Np–O distance (8.0 to 1.5 Å), using these wavefunctions as an initial guess for all the spin multiplicities considered (S = 0–4). The wavefunctions of each spin multiplicity were state-averaged (SA-CASSCF) separately. The numbers of A_1_, B_1_, B_2_, A_2_ states averaged together are 3, 2, 2, 2 for S = 0; 3, 3, 3, 4 for S = 1–3; and 2, 3, 3, 3 for S = 4. Both the valence and subvalence electrons (6*s*, 6*p* of Np and 2*s* of O) are correlated at the MRCI (LC-MRCI). The DKH3 and SO effects were also considered here for distances around equilibrium (1.65–1.80 Å). The cc-pwCVTZ-DK3 and aug-cc-pVTZ basis sets of neptunium and oxygen were utilized [25,26]. Oxygen was supplied with an additional series of diffuse functions (aug-) to account for its anionic nature. All multi-reference calculations were done using the MOLPRO 2021.3 suite of codes, and the C_2v_ point group symmetry elements were exploited.

The calculations on the molecular coordination complexes and their reaction with methane or methanol were performed with Gaussian 16 [27]. Density functional theory (DFT) with the B3LYP functional [28,29] was used to optimize all molecular structures and transition states. The Stuttgart basis set combined with the relativistic small core (RSC) effective core potential was used for neptunium [30], the cc-pVDZ set for hydrogen and carbon, and aug-cc-pVDZ for oxygen and nitrogen [25,26]. The RSC replaces 60 electrons (1*s*–4*f*) with the effective core potential. Harmonic frequency calculations confirmed that all local minima have only real frequencies and that all transition states have one imaginary frequency. Single-point energy calculations were finally done with the MN15 [31] functional to more accurately describe the non-covalent interactions. Solvent effects were also considered via the SMD model for a polar (water) and a non-polar (toluene) solvent.

## 4. Conclusions

This high-level electronic structure study targeted the experimentally synthesized neptunium oxide complex with the goal of understanding the stability of the unusual Np^5+^ oxidation state and assessing the efficiency of the complex in activating methane. We first started with constructing potential energy curves for the “naked” NpO^3+^ species, which revealed the existence of various minima (Np^3+^O, Np^4+^O^−^, Np^5+^O2^−^) depending on the Np–O distance. All minima were found to be metastable with respect to the lowest energy Np^2+^ + O^+^ asymptote. However, the presence of at least one NH_2_^−^ ligand secures the stability of an in situ Np^5+^O^2−^ unit. The ligation of two more NH_2_^−^ anions stabilizes it further, while the additional axial NH_3_ unit does not contribute substantially. Therefore, the stability of the experimentally synthesized neptunium oxide complex can be attributed to the -NR^−^ moiety of the tetradentate N(CH_2_CH_2_NR^−^)_3_ ligand (R = Si^i^Pr_3_). The role of the central nitrogen atom of the ligand serves mostly as anchor. Finally, we demonstrated that the strong bond between neptunium and oxygen prevents the oxidation of methane to methanol unless it is facilitated by a high-spin state with radical character on the NR_2_ moieties, which drives the electron transfer from methane and induces a proton transfer from methane to oxygen. Future work will focus on different *f*-block metals and monitor the stability and reactivity towards methane activation across the lanthanide and actinide series.

## Figures and Tables

**Table 1:** Wavefunction information for the lowest lying electronic states of NpO^3+^ at the equilibrium distance of 1.7 Å.

State ^a^	Coef. ^b^	σ	π_x_	π_y_	δ_+_	δ_−_	φ_+_	φ_−_
1^3^H (^3^B_1_)	0.67	2	2	2	α		α	
	−0.67	2	2	2		α		α
1^3^Σ^−^ (^3^A_2_)	0.74	2	2	2	α	α		
	0.59	2	2	2			α	α
1^3^Π (^3^B_1_)	0.66	2	2	2	α		α	
	0.66	2	2	2		α		α
1^1^Σ^+^ (^1^A_1_)	0.49	2	2	2	2			
	0.49	2	2	2		2		
	−0.46	2	2	2			2	
	−0.46	2	2	2				2
1^1^Π (^1^B_1_)	0.47	2	2	2	β		α	
	−0.47	2	2	2	α		β	
	−0.47	2	2	2		β		α
	0.47	2	2	2		α		β
1^1^Γ (^1^A_2_)	0.67	2	2	2	α	β		
	−0.67	2	2	2	β	α		
(^1^A_1_)	0.67	2	2	2	2			
	−0.67	2	2	2		2		
2^3^Σ^−^ (^3^A_2_)	0.74	2	2	2			α	α
	−0.59	2	2	2	α	α		
1^1^I (^1^A_2_)	−0.66	2	2	2			α	β
	0.66	2	2	2			β	α
(^1^A_1_)	−0.66	2	2	2			2	
	0.66	2	2	2				2
1^1^H (^1^B_1_)	−0.47	2	2	2	β		α	
	0.47	2	2	2	α		β	
	−0.47	2	2	2		β		α
	0.47	2	2	2		α		β

^a^ The electronic term in the C_∞v_ (C_2v_) point group symmetry is listed. Only the B_1_ component of the Π and Η states is included. The corresponding B_2_ components can be obtained by combining δ_+_φ_−_ and δ_−_φ_+_ configurations instead of δ_+_φ_+_ and δ_−_φ_−_. ^b^ This column tabulates the coefficient for each electronic configuration, and the relative molecular orbitals (σ, π_x,y_, δ_±_, φ_±_) are depicted in Figure 3. The π_x,y_*, σ* orbitals are not populated in any of these states and are not included in this table.

**Table 2:** Equilibrium bond lengths r_e_ (Å), harmonic vibrational frequencies ω_e_ (cm^−1^), excitation energies ΔE (eV), and ^2S+1^Λ composition for the lowest energy SO states of NpO^3+^.

State	Composition	r_e_	ω_e_	ΔE
Ω = 4	97% (1^3^H) + 3% (1^1^Γ)	1.729	849	0.000
Ω = 0^+^	59% (1^3^Σ^−^) + 25% (1^3^Π) + 16% (1^1^Σ^+^)	1.732	831	0.383
Ω = 1	48% (1^3^Π) + 33% (1^3^Σ^−^) + 19% (1^1^Π)	1.728	844	0.633
Ω = 5	99% (1^3^H) + 1% (1^1^H)	1.730	843	0.878
Ω = 0^−^	100% (1^3^Π)	1.734	821	1.298
Ω = 1	64% (1^3^Σ^−^) + 21% (1^1^Π) + 13% (1^3^Π) + 2% (2^3^Σ^−^)	1.730	841	1.391
Ω = 0^+^	66% (1^3^Π) + 27% (1^1^Σ^+^) + 6% (1^3^Σ^−^) + 1% (2^3^Σ^−^)	1.726	851	1.504
Ω = 6	91% (1^3^H) + 9% (1^1^Ι)	1.735	815	1.563
Ω = 2	100% (1^3^Π)	1.734	823	1.623
Ω = 1	50% (2^3^Σ^−^) + 25% (1^3^Π) + 22% (1^1^Π) + 3% (1^3^Σ^−^)	1.730	842	2.289
Ω = 0^+^	37% (2^3^Σ^−^) + 31% (1^1^Σ^+^) + 26% (1^3^Σ^−^) + 6% (1^3^Π)	1.724	857	2.337
Ω = 4	97% (1^1^Γ) + 3% (1^3^H)	1.709	886	2.491
Ω = 0^+^	63% (2^3^Σ^−^) + 25% (1^1^Σ^+^) + 9% (1^3^Σ^−^) + 3% (1^3^Π)	1.736	834	2.741
Ω = 1	48% (2^3^Σ^−^) + 38% (1^1^Π) + 14% (1^3^Π)	1.733	841	2.948
Ω = 6	91% (1^1^Ι) + 9% (1^3^H)	1.743	822	2.954
Ω = 5	99% (1^1^H) + 1% (1^3^H)	1.724	868	3.035

## Data Availability

Data are contained within the article or Supplementary Materials.

## Supplementary Materials

The following supporting information can be downloaded at: https://www.mdpi.com/article/10.3390/molecules31081258/s1, Figure S1: LC-MRCI-DKH3 PECs of NpO^3+^ as a function of the Np–O interatomic distance R_Np–O_ for the various spin multiplicities. Figure S2: CASSCF-DKH3 PECs of NpO^3+^ as a function of the Np–O interatomic distance R_Np–O_ for the various spin multiplicities. Figure S3: Contours of the molecular orbitals of NpO^3+^ around the higher energy local minimum at a distance of 2.7 Å. Figure S4: Spin–orbit PECs of NpO^3+^ as a function of the Np–O interatomic distance R_Np–O_ for the various spin multiplicities. Figure S5: B3LYP energy diagram for the reaction of N(CH_2_CH_2_NH)_3_NpO + CH_4_ towards N(CH_2_CH_2_NH)_3_Np + CH_3_OH. [NpO] and [Np] denote N(CH_2_CH_2_NH)_3_NpO and N(CH_2_CH_2_NH)_3_Np. Red/black/blue/green lines correspond to S = 1/S = 0/S = 2/S = 3, and solid/dashed horizontal lines to the radical/2+2 mechanisms. Figure S6: MN15//B3LYP energy diagram for the reaction of N(CH_2_CH_2_NH)_3_NpO + CH_4_ towards N(CH_2_CH_2_NH)_3_Np + CH_3_OH. [NpO] and [Np] denote N(CH_2_CH_2_NH)_3_NpO and N(CH_2_CH_2_NH)_3_Np. Red/black/blue/green lines correspond to S = 1/S = 0/S = 2/S = 3, and solid/dashed horizontal lines to the radical/2+2 mechanisms. Figure S7: MN15//B3LYP + SMD (water) energy diagram for the reaction of N(CH_2_CH_2_NH)_3_NpO + CH_4_ towards N(CH_2_CH_2_NH)_3_Np + CH_3_OH. [NpO] and [Np] denote N(CH_2_CH_2_NH)_3_NpO and N(CH_2_CH_2_NH)_3_Np. Red/black/blue/green lines correspond to S = 1/S = 0/S = 2/S = 3, and solid/dashed horizontal lines to the radical/2+2 mechanisms. Figure S8: MN15//B3LYP + SMD (toluene) energy diagram for the reaction of N(CH_2_CH_2_NH)_3_NpO + CH_4_ towards N(CH_2_CH_2_NH)_3_Np + CH_3_OH. [NpO] and [Np] denote N(CH_2_CH_2_NH)_3_NpO and N(CH_2_CH_2_NH)_3_Np. Red/black/blue/green lines correspond to S = 1/S = 0/S = 2/S = 3, and solid/dashed horizontal lines to the radical/2+2 mechanisms. Figure S9: Singly occupied orbitals of the ECR and IC structures of the S = 2 state. Table S1: Cartesian coordinates (Å) of (NH_3_)_1_(NH_2_^−^)_3_Np^5+^O^2−^ used for all (NH_3_)_x_(NH_2_^−^)_y_Np^5+^O^2−^ species (x = 0–1, y = 0–3) by removing NH_3_ or one or more NH_2_^−^ ligands as necessary. Table S2: Energy ΔE (eV) and composition of the eighteen spin–orbit states of the N(CH_2_CH_2_NH)_3_NpO complex in terms of the first six spin–orbit free triplet states. Table S3: Cartesian coordinates (Å) of all intermediate and transition states for the reaction N(CH_2_CH_2_NH)_3_NpO (S = 0, 1) + CH_4_ and for both [2+2] and radical mechanisms. Table S4: Cartesian coordinates (Å) of all intermediate and transition states for the reaction N(CH_2_CH_2_NH)_3_NpO (S = 2, 3) + CH_4_ and for both [2+2] and radical mechanisms.
