# Supplementary material for: Electronic Structure, Ligand Effects, and Chemical Reactivity of the Ground and Low-Lying Excited Electronic States of NpO3+
Source: Molecules. 2026 Apr 10;31(8):1258. doi: 10.3390/molecules31081258 (PMC13118823; doi:10.3390/molecules31081258)
Supplement: Supplementary file 1 [file molecules-31-01258-s001.zip › molecules-4196084-supplementary.pdf]

Supporting Information

Electronic structure, ligand effects, and chemical reactivity of the ground and low-lying excited electronic states of NpO^{3+}

*Taylor Gregory and Evangelos Miliordos**

Department of Chemistry and Biochemistry, Auburn University, Auburn, AL 36849-5312, USA

Corresponding Author

* E-mail: emiliord@auburn.edu

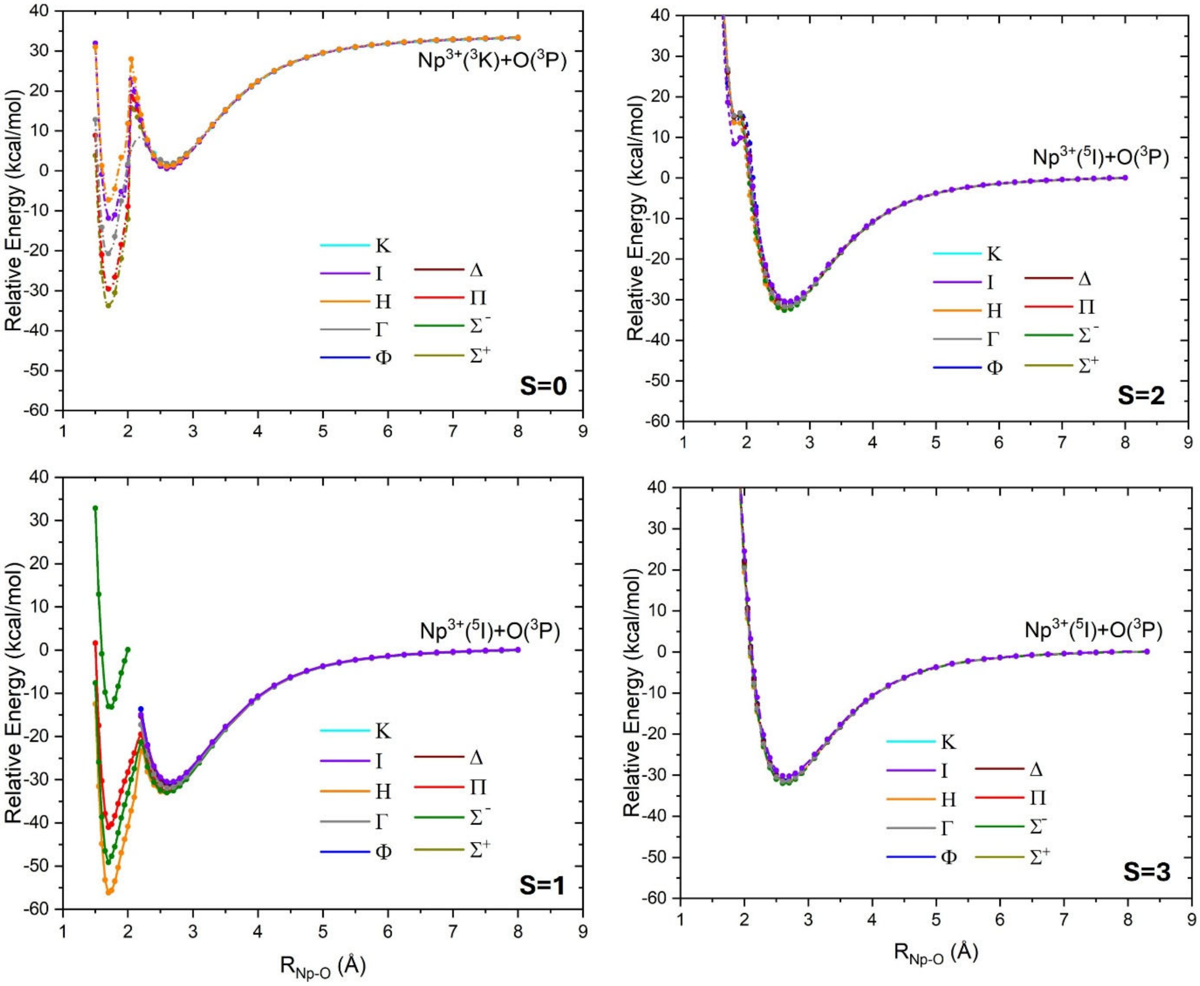

Figure S1. LC-MRCI-DKH3 PECs of NpO^{3+} as a function of the Np-O interatomic distance $R_{Np\text{-}O}$ for the various spin multiplicities.

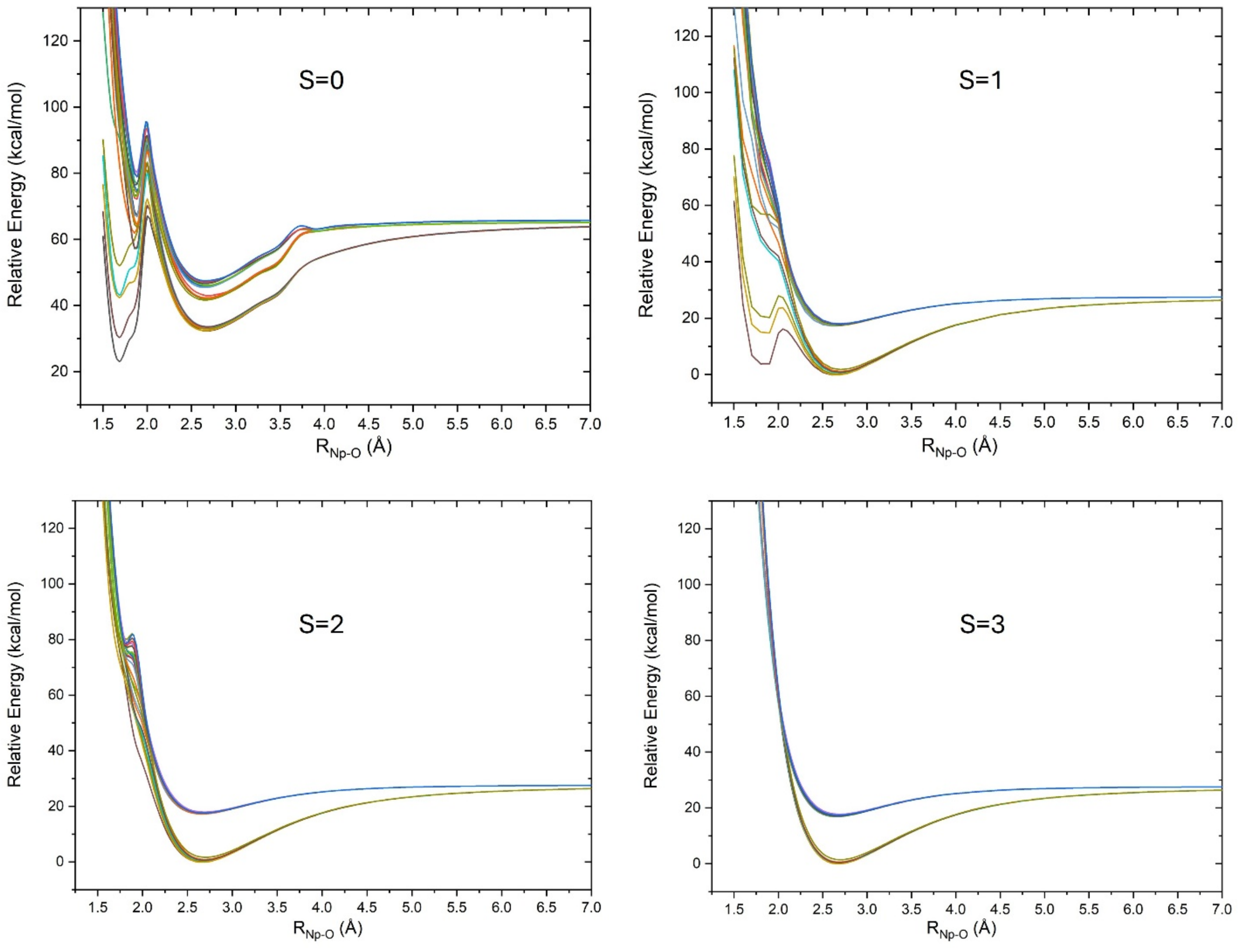

Figure S2. CASSCF-DKH3 PECs of NpO^{3+} as a function of the Np-O interatomic distance R_{Np-O} for the various spin multiplicities.

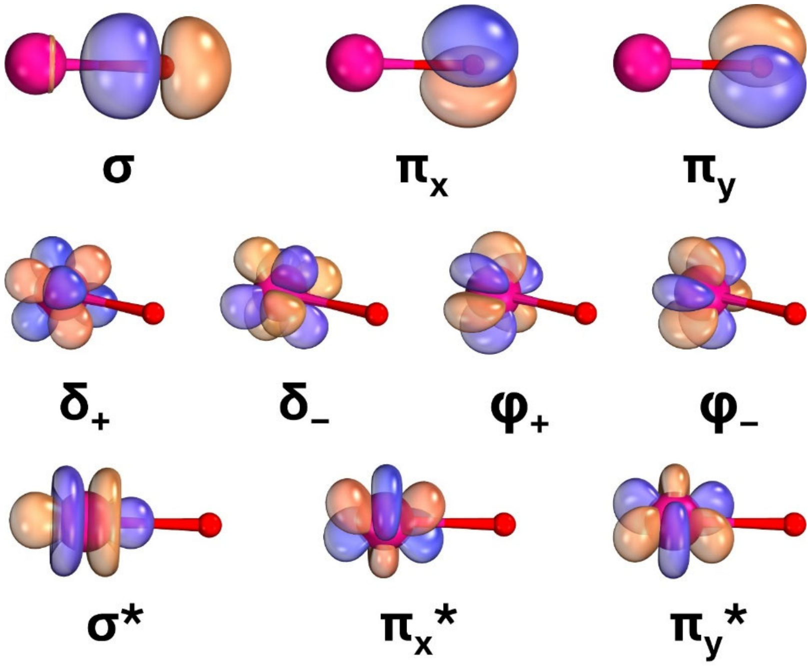

Figure S3. Contours of the molecular orbitals of NpO^{3+} around the higher energy local minimum at distance of 2.7 Å.

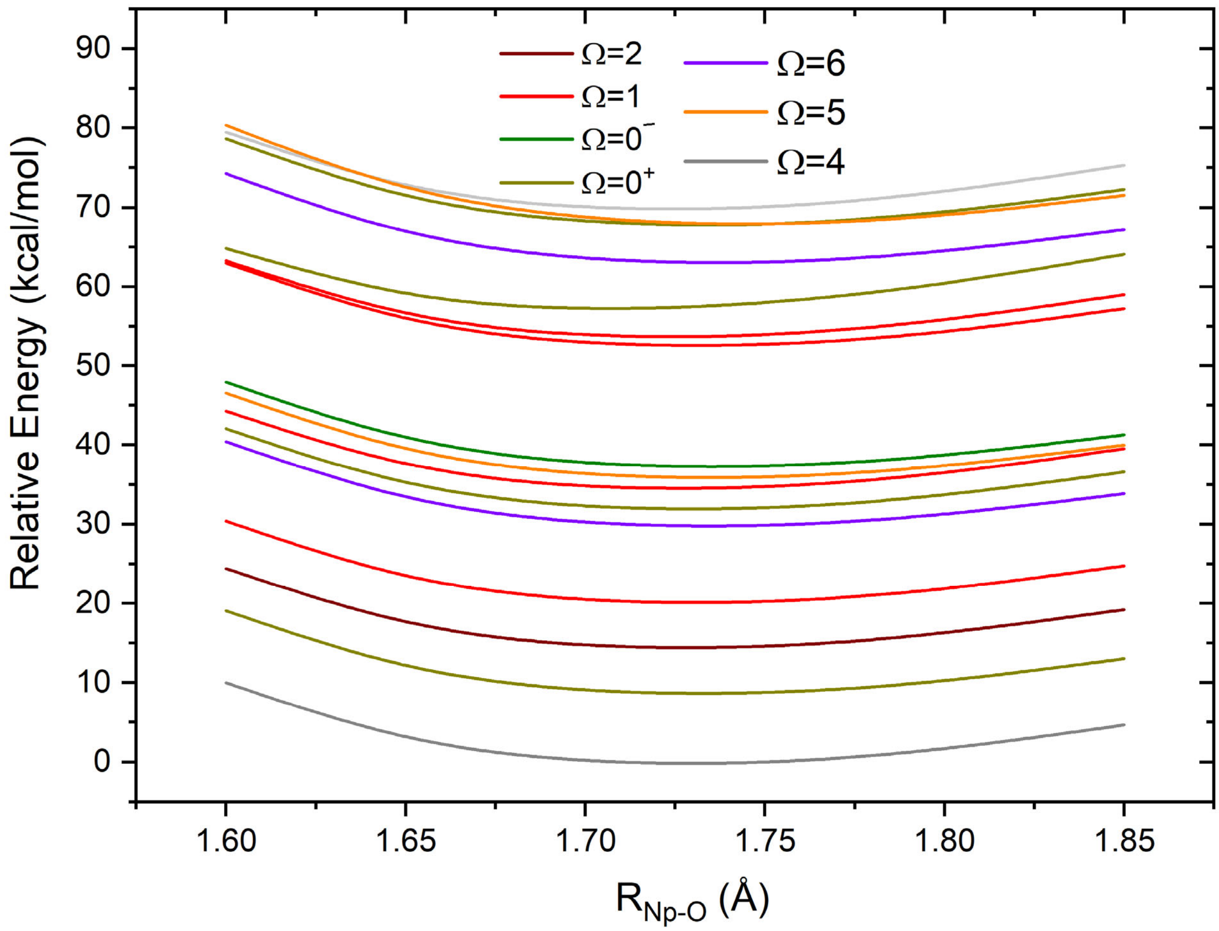

Figure S4. Spin-orbit PECs of NpO^{3+} as a function of the Np-O interatomic distance R_{Np-O} for the various spin multiplicities.

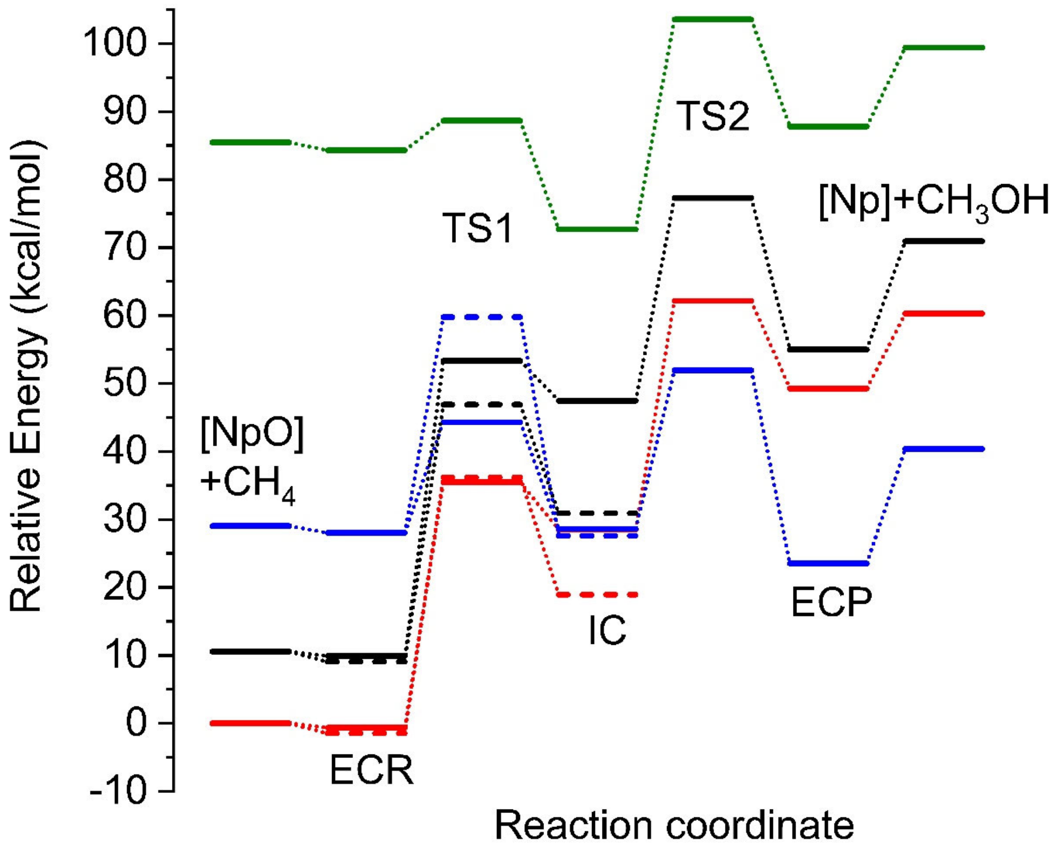

Figure S5. B3LYP energy diagram for the reaction of $N(CH_2CH_2NH)_3NpO$ + CH_4 towards $N(CH_2CH_2NH)_3Np$ + CH_3OH. [NpO] and [Np] denote $N(CH_2CH_2NH)_3NpO$ and $N(CH_2CH_2NH)_3Np$. Red/black/blue/green lines correspond to S=1/S=0/S=2/S=3, and solid/dashed horizontal lines to the radical/2+2 mechanisms.

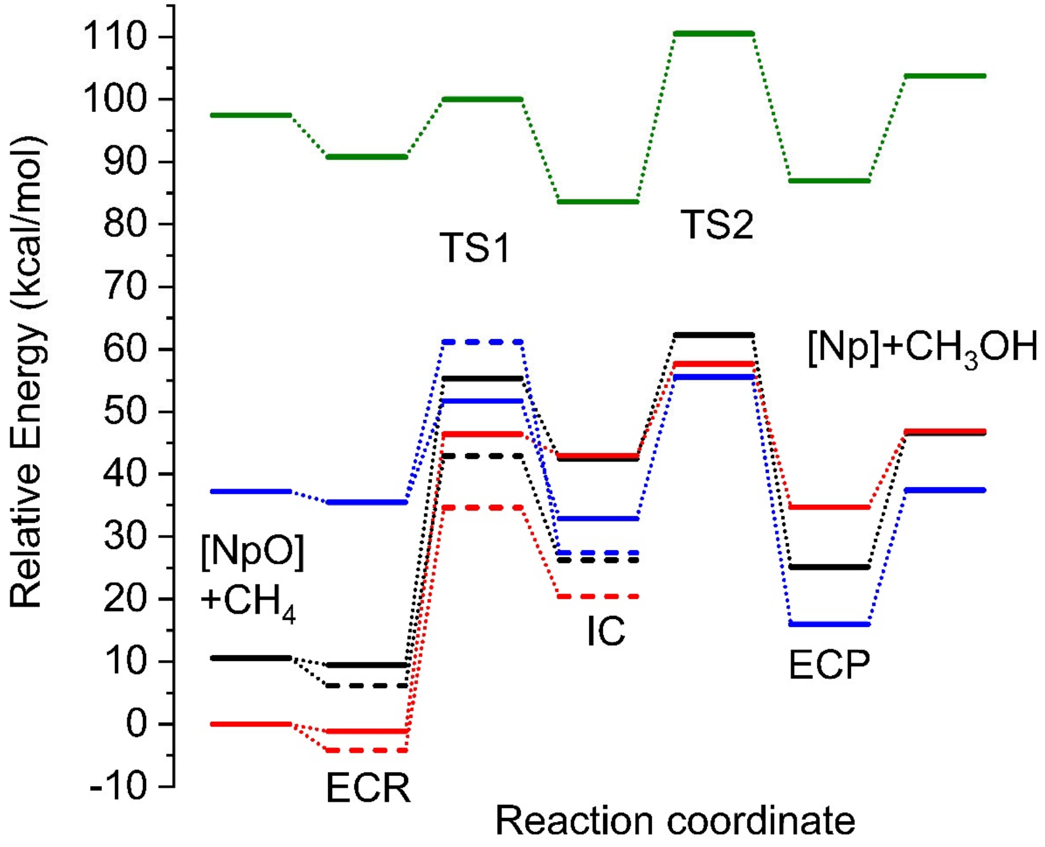

Figure S6. MN15//B3LYP energy diagram for the reaction of $N(CH_2CH_2NH)_3NpO + CH_4$ towards $N(CH_2CH_2NH)_3Np + CH_3OH$. [NpO] and [Np] denote $N(CH_2CH_2NH)_3NpO$ and $N(CH_2CH_2NH)_3Np$. Red/black/blue/green lines correspond to S=1/S=0/S=2/S=3, and solid/dashed horizontal lines to the radical/2+2 mechanisms.

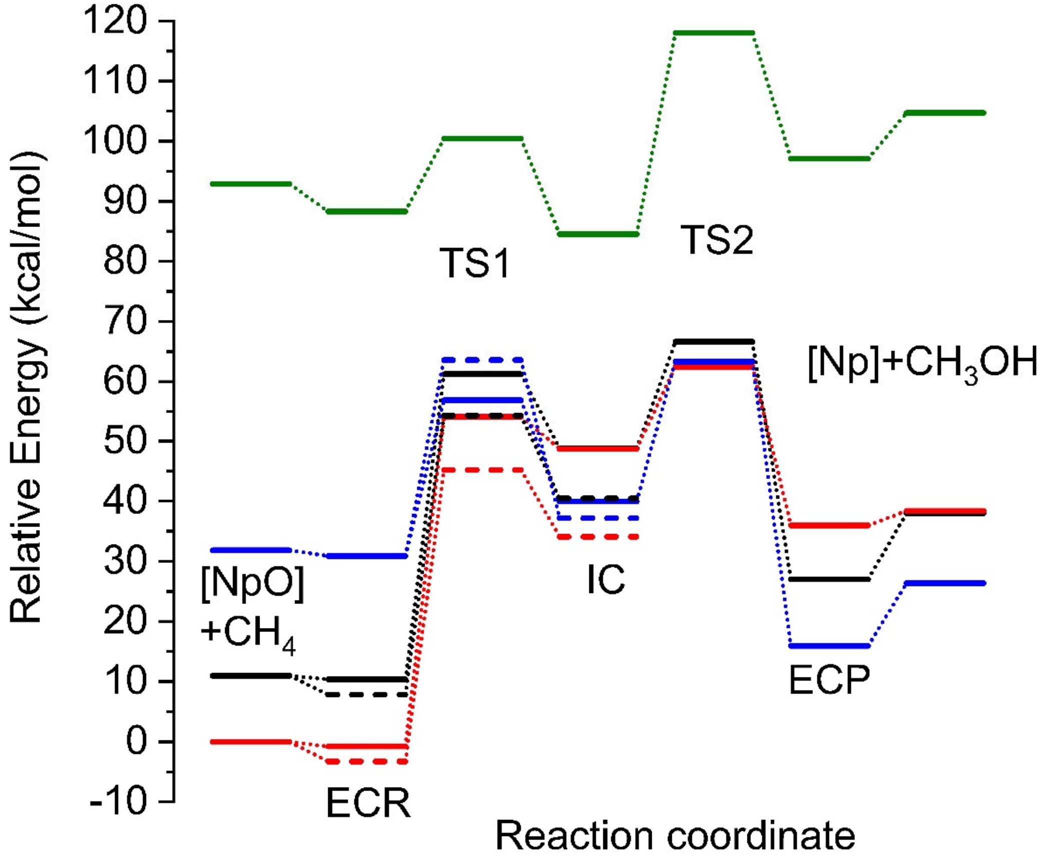

Figure S7. MN15//B3LYP + SMD (water) energy diagram for the reaction of $N^-(CH_2CH_2NH)_3NpO + CH_4$ towards $N(CH_2CH_2NH)_3Np + CH_3OH$. [NpO] and [Np] denote $N^-(CH_2CH_2NH)_3NpO$ and $N(CH_2CH_2NH)_3Np$. Red/black/blue/green lines correspond to S=1/S=0/S=2/S=3, and solid/dashed horizontal lines to the radical/2+2 mechanisms.

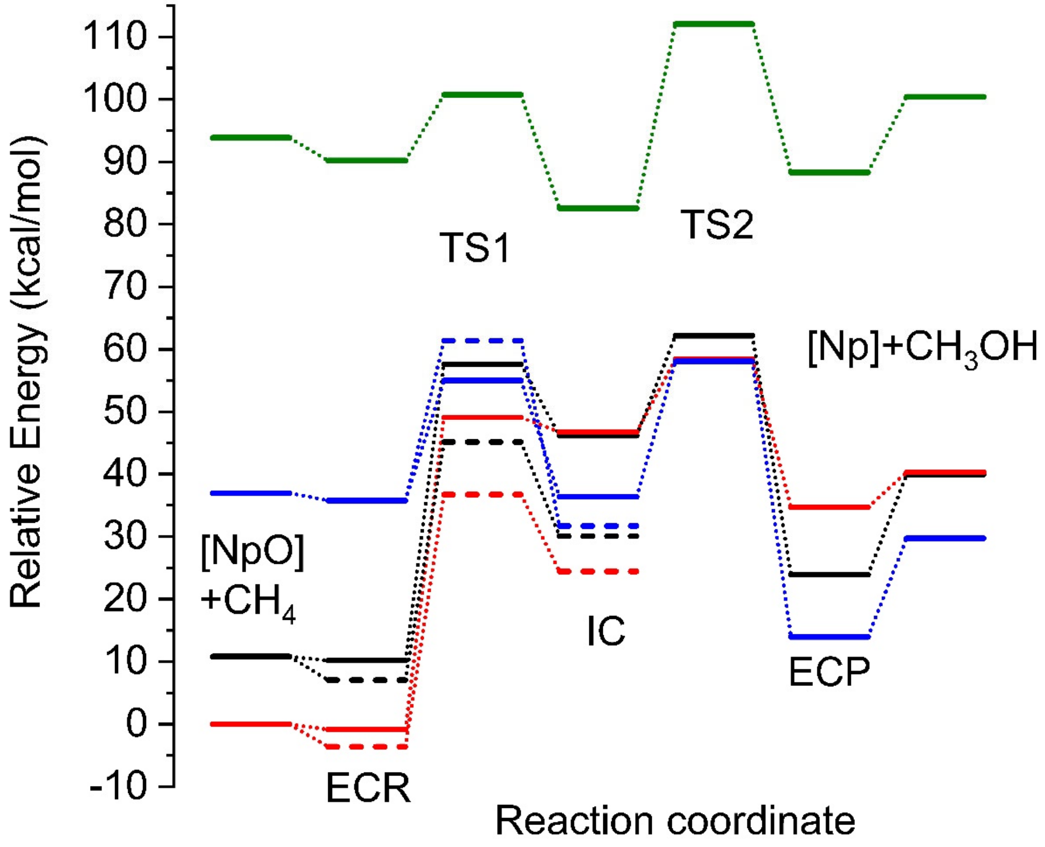

Figure S8. MN15//B3LYP + SMD (toluene) energy diagram for the reaction of $N^-(CH_2CH_2NH)_3NpO$ + CH_4 towards $N(CH_2CH_2NH)_3Np$ + CH_3OH. [NpO] and [Np] denote $N^-(CH_2CH_2NH)_3NpO$ and $N(CH_2CH_2NH)_3Np$. Red/black/blue/green lines correspond to S=1/S=0/S=2/S=3, and solid/dashed horizontal lines to the radical/2+2 mechanisms.

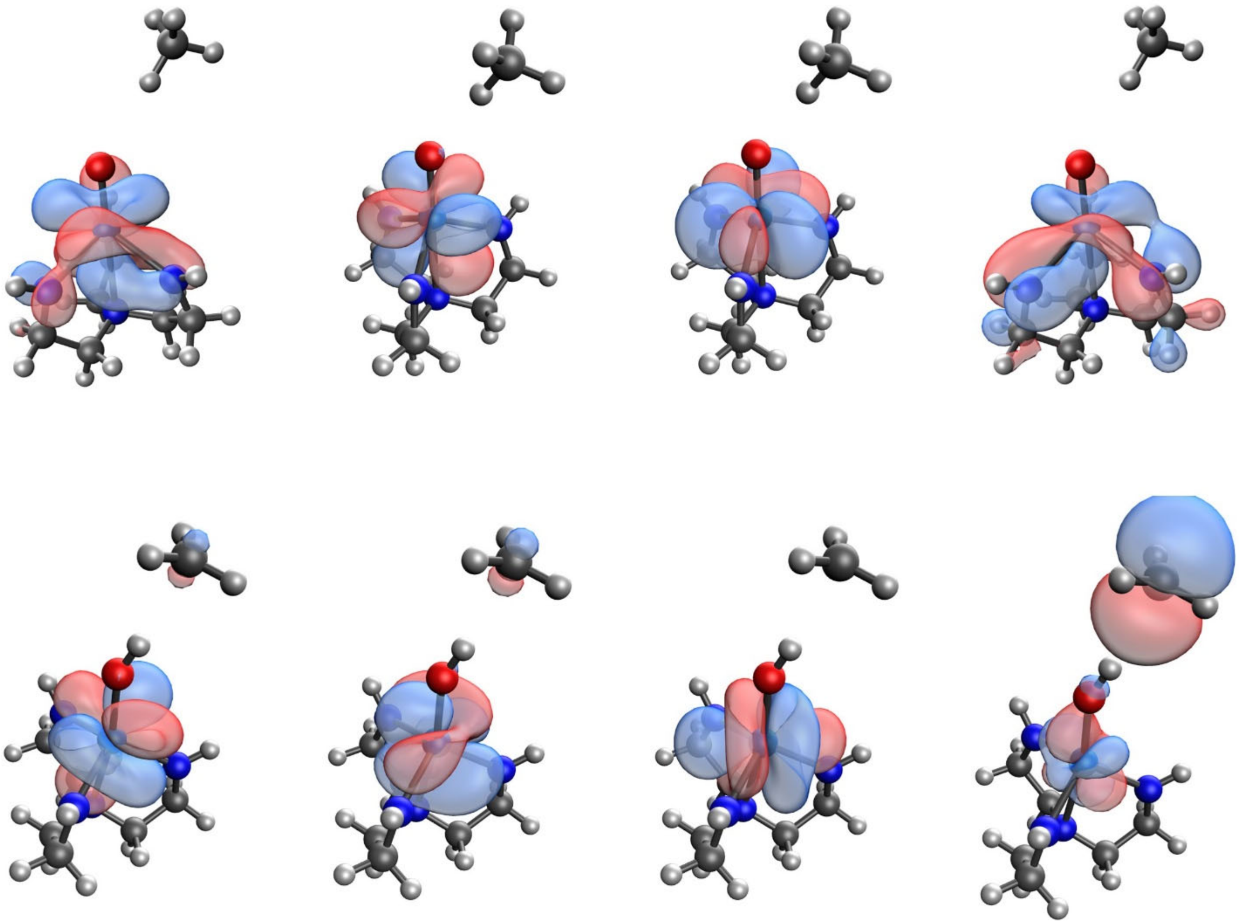

Figure S9. Singly occupied orbitals of the ECR and IC structures of the S=2 state.

Table S1. Cartesian coordinates (Å) of $(NH_3)_1(NH_2^-)_3Np^{5+}O^{2-}$ used for all $(NH_3)_x(NH_2^-)_yNp^{5+}O^{2-}$ species (x=0-1, y=0-3) species by removing NH_3 or one or more NH_2^- ligands as necessary.

N	-2.427295	-0.000000	-0.000000
N	-0.494728	-1.744822	-1.216800
N	-0.494728	1.926190	-0.902660
Np	0.244562	0.000000	0.000000
N	-0.494728	-0.181369	2.119460
O	2.130940	0.000000	0.000000
H	-2.783958	0.086012	-1.005134
H	-2.783963	0.827464	0.577054
H	-2.783964	-0.913475	0.428078
H	0.229783	2.685274	-1.258385
H	-1.582973	2.114801	-0.991048
H	0.229783	-0.252843	2.954708
H	-1.582973	-0.199128	2.326996
H	0.229783	-2.432430	-1.696322
H	-1.582973	-1.915674	-1.335948

Table S2. Energy ΔE (eV) and composition of eighteen spin-orbit states of the $N(CH_2CH_2NH)_3NpO$ complex in terms of the first six spin-orbit free triplet states.

States		Spin-orbit States																	
State	M_S	1	2	3	4	5	6	7	8	9	10	11	12	13	14	15	16	17	18
1	1	22.1	21.9	1.3	2.0	0.2	0.1	0.0	3.0	0.3	0.8	0.5	0.5	1.2	2.0	24.7	6.7	10.6	2.1
2	1	21.7	21.6	1.5	1.3	1.3	0.1	0.0	2.5	0.3	1.4	2.7	0.2	3.0	0.0	17.4	7.6	15.4	2.1
3	1	0.3	1.6	0.1	20.8	29.8	3.5	3.8	0.0	0.4	1.6	3.1	3.3	4.9	17.9	0.7	5.6	2.4	0.3
4	1	0.9	1.0	13.1	0.1	0.0	0.0	1.6	24.0	6.4	17.3	0.9	4.9	4.9	0.3	0.3	0.1	6.0	18.0
5	1	0.7	1.3	9.4	0.0	0.4	6.4	1.8	13.4	18.3	5.5	0.3	19.0	7.4	3.4	0.0	7.2	0.1	5.6
6	1	0.0	0.0	0.0	0.9	1.9	3.5	4.3	0.7	10.7	26.0	18.6	9.9	0.2	1.5	0.4	3.9	10.7	6.9
1	0	0.0	0.6	0.1	13.9	0.4	0.0	67.3	0.1	0.0	0.6	0.0	8.7	0.0	4.6	0.8	0.0	0.1	2.8
2	0	0.2	0.0	0.0	0.4	13.9	63.0	0.0	5.7	1.0	0.1	6.5	0.0	0.2	0.0	0.0	5.4	3.6	0.0
3	0	0.0	0.2	49.3	0.0	0.0	0.0	0.0	0.0	0.0	1.9	0.1	5.0	0.0	7.5	9.7	0.3	0.0	26.1
4	0	0.1	5.3	0.0	24.9	0.4	0.0	11.5	0.0	0.0	0.7	0.1	10.5	0.1	43.1	2.1	0.0	0.0	1.2
5	0	3.5	0.1	0.0	0.4	25.3	10.1	0.0	0.4	2.6	0.0	5.4	0.3	30.3	0.1	0.1	15.7	5.7	0.0
6	0	4.4	0.0	0.0	0.2	2.7	0.0	0.0	0.1	23.8	0.0	29.9	0.0	24.1	0.0	0.1	13.3	1.5	0.0
1	-1	21.3	22.6	0.9	1.0	1.2	0.2	0.0	4.3	0.3	0.2	0.4	1.3	1.4	0.6	16.6	13.0	13.2	1.7
2	-1	22.5	20.8	2.0	2.3	0.1	0.0	0.0	1.9	0.3	1.0	2.5	0.9	2.7	0.3	24.4	3.1	12.8	2.6
3	-1	0.6	0.7	0.0	29.9	21.2	4.8	2.6	0.0	0.9	0.8	5.5	1.2	6.6	13.3	1.3	7.6	2.5	0.4
4	-1	1.1	0.9	11.3	0.1	0.0	0.1	0.4	30.1	7.0	13.4	1.6	3.0	6.8	0.5	0.0	1.0	9.0	13.7
5	-1	0.6	1.5	10.8	0.0	0.3	4.5	2.7	13.4	13.0	6.2	1.2	24.0	6.2	3.4	0.4	6.5	0.5	4.9
6	-1	0.0	0.0	0.0	1.9	0.9	3.7	3.9	0.5	14.9	22.6	20.8	7.5	0.1	1.6	1.0	3.2	6.0	11.6
ΔE		0.000	0.053	0.129	0.407	0.427	0.730	0.730	0.827	1.042	1.122	1.295	1.360	1.447	1.506	1.530	1.539	1.587	1.664

Table S3. Cartesian coordinates (Å) of all intermediate and transition states for the reaction $N(CH_2CH_2NH)_3NpO$ (S=0,1) + CH_4 and for both [2+2] and radical mechanisms obtained at the B3LYP/RSC level of theory.

Species	S=0				S=1			
ECR	N	0.457362	1.239215	-1.637160	N	-0.404217	1.000180	1.787637
	C	1.834096	1.092111	-2.078877	C	-1.759005	0.783351	2.264637
	C	2.699577	0.626781	-0.905956	C	-2.671753	0.485839	1.073459
	N	2.045360	-0.454661	-0.130381	N	-2.046543	-0.468020	0.125923
	C	2.032694	-1.747093	-0.854971	C	-2.010090	-1.851053	0.657180
	C	0.935484	-2.664360	-0.307138	C	-0.942034	-2.681760	-0.059423
	H	1.271907	-3.139746	0.639848	H	-1.320609	-3.019473	-1.048878
	H	0.809001	-3.497359	-1.025462	H	-0.792225	-3.607680	0.529191
	N	-0.279913	-1.883976	-0.140753	N	0.270960	-1.887799	-0.165662
	H	-1.113500	-2.460697	-0.264482	H	1.102689	-2.480191	-0.177069
	H	3.030856	-2.229554	-0.811620	H	-3.011875	-2.322683	0.586435
	H	1.808361	-1.528150	-1.908029	H	-1.742525	-1.783964	1.720707
	C	2.636442	-0.593632	1.218920	C	-2.687700	-0.413700	-1.206556
	H	2.257707	-1.532949	1.644926	H	-2.331666	-1.283537	-1.775644
	H	3.741102	-0.669967	1.162722	H	-3.789990	-0.495327	-1.119921
	C	2.200977	0.564422	2.116175	C	-2.277782	0.858719	-1.947115
	H	2.391311	0.275142	3.169285	H	-2.516387	0.723935	-3.021274
	N	0.795498	0.813205	1.847531	N	-0.859229	1.059007	-1.709281
	H	0.434123	1.561487	2.452075	H	-0.510896	1.872796	-2.230968
	H	2.830296	1.459913	1.930811	H	-2.888416	1.721627	-1.608084
	H	3.702967	0.306122	-1.253137	H	-3.659647	0.110077	1.409699
	H	2.841417	1.473402	-0.219412	H	-2.842500	1.421383	0.522278
	H	1.925261	0.376350	-2.924049	H	-1.815261	-0.046460	3.002004
	H	2.272503	2.041579	-2.451103	H	-2.180650	1.667508	2.786784
	H	-0.125024	1.597156	-2.397102	H	0.208357	1.274927	2.558288
	C	-5.806423	-0.589683	0.084152	C	5.841880	-0.574659	-0.069880
	H	-6.402704	0.322328	0.238812	H	6.178337	-0.062649	-0.984324
	H	-5.849987	-1.210663	0.992010	H	5.958980	-1.661530	-0.197853
	H	-6.222129	-1.155667	-0.763141	H	6.456391	-0.241026	0.779861
	H	-4.762373	-0.316906	-0.130845	H	4.785389	-0.335255	0.123038
	Np	-0.509923	0.293938	0.115941	Np	0.507241	0.308312	-0.110694
	O	-2.217936	0.358028	-0.509143	O	2.231397	0.288812	0.487020
TS1/[2+2]	N	-0.338337	-1.489598	1.369793	N	-0.203146	-0.975040	1.768488
	C	-1.760860	-1.742051	1.535395	C	-1.603350	-1.184542	2.099400
	C	-2.543150	-0.491275	1.136108	C	-2.452991	-0.127873	1.394305
	N	-2.010402	0.110198	-0.105129	N	-2.025364	0.068879	-0.006925
	C	-2.384694	-0.640202	-1.320435	C	-2.464524	-1.015136	-0.908027
	C	-1.418103	-0.327569	-2.467250	C	-1.598387	-1.048919	-2.172025
	H	-1.674870	0.649652	-2.928982	H	-1.923701	-0.257538	-2.880334
	H	-1.582585	-1.081451	-3.260710	H	-1.795299	-2.006965	-2.689396
	N	-0.055542	-0.355886	-1.955944	N	-0.199698	-0.907503	-1.792727

	H	0.605818	-0.637570	-2.683104	H	0.408896	-1.428230	-2.428283
	H	-3.436566	-0.435915	-1.611419	H	-3.541944	-0.917878	-1.159496
	H	-2.305437	-1.710482	-1.081267	H	-2.331582	-1.964027	-0.368353
	C	-2.355020	1.541477	-0.207002	C	-2.415780	1.399314	-0.512767
	H	-2.129120	1.863129	-1.233117	H	-2.292359	1.388375	-1.604319
	H	-3.437545	1.710906	-0.032852	H	-3.482225	1.616927	-0.298382
	C	-1.502347	2.362481	0.761119	C	-1.503225	2.479249	0.067972
	H	-1.548697	3.424936	0.451694	H	-1.616806	3.397089	-0.541683
	N	-0.150736	1.833389	0.714311	N	-0.144255	1.968593	0.042335
	H	0.517163	2.455047	1.184079	H	0.542541	2.698230	0.265066
	H	-1.927174	2.326499	1.785772	H	-1.823190	2.754500	1.094203
	H	-3.625936	-0.713583	1.041196	H	-3.531740	-0.380971	1.452506
	H	-2.430513	0.257196	1.932985	H	-2.312808	0.831266	1.912953
	H	-2.091915	-2.611035	0.929133	H	-1.942367	-2.202015	1.810660
	H	-2.030732	-1.989806	2.582428	H	-1.801553	-1.104220	3.187965
	H	0.206297	-2.315673	1.634922	H	0.384564	-1.646958	2.269827
	C	3.029577	1.222043	-0.084133	C	3.101280	1.131520	-0.022422
	H	3.009936	1.726396	0.901417	H	2.962601	1.751378	0.884918
	H	2.706489	1.953857	-0.847894	H	2.926190	1.774615	-0.905956
	H	4.086710	0.981830	-0.295169	H	4.165629	0.839093	-0.045199
	H	2.830568	-0.340543	-0.179498	H	2.832263	-0.429429	-0.018824
	Np	0.728919	-0.076955	0.073393	Np	0.733445	-0.048827	0.003894
	O	2.245167	-1.324087	-0.174898	O	2.198948	-1.382311	-0.007106
IC/[2+2]	N	-0.317985	1.963764	-0.381526	N	-0.337583	-2.005050	0.077051
	C	-1.729394	2.298908	-0.324719	C	-1.754317	-2.316207	0.037514
	C	-2.533317	1.035908	-0.627742	C	-2.525066	-1.100191	0.550939
	N	-1.972361	-0.123868	0.096670	N	-1.973792	0.144615	-0.024029
	C	-2.327819	-0.098803	1.531337	C	-2.385779	0.322493	-1.432678
	C	-1.307097	-0.866421	2.372417	C	-1.387293	1.187531	-2.202478
	H	-1.498954	-1.959193	2.303329	H	-1.561201	2.262797	-1.980206
	H	-1.480214	-0.601272	3.435029	H	-1.603094	1.069677	-3.283829
	N	0.028132	-0.519464	1.915881	N	-0.041127	0.772539	-1.846648
	H	0.733551	-0.837094	2.586633	H	0.643658	1.192324	-2.482382
	H	-3.355426	-0.483699	1.695022	H	-3.412597	0.737360	-1.499357
	H	-2.314183	0.952887	1.850806	H	-2.402502	-0.674963	-1.894012
	C	-2.317482	-1.401499	-0.556661	C	-2.273823	1.322936	0.813126
	H	-2.016582	-2.208332	0.127748	H	-1.984247	2.213294	0.235471
	H	-3.412480	-1.488731	-0.719662	H	-3.361016	1.399939	1.026179
	C	-1.546561	-1.559331	-1.871245	C	-1.458303	1.292900	2.110275
	H	-1.609675	-2.623063	-2.174199	H	-1.505818	2.304879	2.559525
	N	-0.176585	-1.125920	-1.654573	N	-0.098300	0.889211	1.795427
	H	0.446220	-1.517443	-2.367777	H	0.539631	1.139852	2.556761
	H	-2.048727	-0.990277	-2.683223	H	-1.939864	0.618791	2.851325
	H	-3.607824	1.177111	-0.391259	H	-3.610596	-1.193507	0.341795
	H	-2.454616	0.824599	-1.702456	H	-2.399401	-1.046099	1.640540
	H	-2.015065	2.717109	0.662686	H	-2.090220	-2.589181	-0.984715

	H	-2.003164	3.070798	-1.071149	H	-2.008358	-3.182234	0.680047
	H	0.286463	2.785691	-0.294222	H	0.254914	-2.829867	-0.050116
	C	2.184068	-1.824534	0.132488	C	2.194321	1.864000	-0.027532
	H	2.792590	-1.859481	-0.790780	H	2.810907	1.810056	0.886293
	H	1.628121	-2.765475	0.251151	H	1.619504	2.799176	-0.058326
	H	2.850585	-1.661074	0.998262	H	2.831693	1.756760	-0.922493
	H	3.480740	1.188829	0.259537	H	3.528916	-1.182235	-0.060449
	Np	0.750594	0.101645	-0.066731	Np	0.750459	-0.116127	0.015530
	O	2.541327	1.244769	0.057508	O	2.569274	-1.232121	-0.014242
TS1/radical	N	-0.140533	1.174564	-1.665978	N	-0.123450	0.916758	-1.821203
	C	-1.516775	1.509775	-1.998311	C	-1.492821	1.241148	-2.190334
	C	-2.455290	0.463344	-1.389115	C	-2.451627	0.301670	-1.452006
	N	-2.073003	0.129360	0.003061	N	-2.075456	0.143269	-0.027301
	C	-2.398060	1.218328	0.953793	C	-2.396969	1.346133	0.776097
	C	-1.559424	1.112369	2.232139	C	-1.560449	1.403661	2.058973
	H	-1.995588	0.347550	2.911216	H	-1.996720	0.728738	2.827588
	H	-1.659284	2.075725	2.771258	H	-1.665638	2.426466	2.472563
	N	-0.182310	0.812709	1.869535	N	-0.179725	1.068059	1.743119
	H	0.456091	1.040913	2.635345	H	0.456655	1.426910	2.458087
	H	-3.483430	1.228945	1.183936	H	-3.482714	1.389394	1.001089
	H	-2.152409	2.166187	0.454825	H	-2.147728	2.221637	0.160095
	C	-2.631027	-1.172464	0.438034	C	-2.645375	-1.089073	0.566375
	H	-2.483418	-1.238239	1.525276	H	-2.509405	-1.014751	1.654685
	H	-3.722472	-1.218145	0.243889	H	-3.735161	-1.153615	0.368073
	C	-1.901823	-2.345664	-0.223388	C	-1.918413	-2.342989	0.071099
	H	-2.178617	-3.263049	0.335254	H	-2.216120	-3.179202	0.736146
	N	-0.471178	-2.077897	-0.199083	N	-0.484699	-2.090916	0.088209
	H	0.051713	-2.919028	-0.465119	H	0.031157	-2.968020	-0.038173
	H	-2.285132	-2.501197	-1.255128	H	-2.286801	-2.623714	-0.939488
	H	-3.511756	0.798541	-1.437929	H	-3.501481	0.648331	-1.544772
	H	-2.373466	-0.462983	-1.975462	H	-2.388359	-0.693939	-1.913898
	H	-1.798796	2.524576	-1.642850	H	-1.750339	2.299192	-1.965756
	H	-1.704781	1.519519	-3.091769	H	-1.686041	1.116662	-3.275675
	H	0.510353	1.777199	-2.174853	H	0.540167	1.401333	-2.429615
	C	5.099005	-0.038259	0.018821	C	5.083748	-0.014859	-0.030089
	H	5.314751	-0.363101	-1.005418	H	5.251212	-0.563495	-0.964411
	H	5.221637	-0.809357	0.788087	H	5.273695	-0.592196	0.882388
	H	5.490258	0.950081	0.285234	H	5.486050	1.004763	-0.019191
	H	3.646260	0.201551	-0.001273	H	3.675994	0.205500	-0.010049
	Np	0.564082	-0.102416	-0.002313	Np	0.564501	-0.110895	0.019649
	O	2.555995	0.364163	-0.014252	O	2.560960	0.362364	0.004057
IC/radical	N	-0.187830	2.022207	0.451555	N	-0.215361	2.082521	0.013443
	C	-1.570405	2.469302	0.556158	C	-1.607304	2.514084	0.029533
	C	-2.468249	1.509606	-0.232235	C	-2.489568	1.398519	-0.542183
	N	-2.112236	0.093404	0.019320	N	-2.117991	0.068036	-0.004123
	C	-2.541466	-0.360256	1.362331	C	-2.550513	-0.108984	1.402165

	C	-1.757598	-1.596681	1.814179	C	-1.741868	-1.201508	2.110565
	H	-2.182842	-2.509104	1.341934	H	-2.128377	-2.204518	1.825292
	H	-1.936571	-1.719032	2.902258	H	-1.948007	-1.106210	3.196309
	N	-0.351665	-1.411639	1.490165	N	-0.331259	-1.039014	1.788912
	H	0.220341	-2.133414	1.936175	H	0.249547	-1.593683	2.422307
	H	-3.634798	-0.548532	1.385722	H	-3.639013	-0.318058	1.456259
	H	-2.331003	0.461257	2.061902	H	-2.369026	0.845502	1.916717
	C	-2.596286	-0.804735	-1.055002	C	-2.581550	-1.036323	-0.876473
	H	-2.485848	-1.834328	-0.686493	H	-2.470633	-1.967292	-0.302407
	H	-3.673843	-0.633452	-1.257677	H	-3.657383	-0.918960	-1.122390
	C	-1.763599	-0.656436	-2.333034	C	-1.732997	-1.148145	-2.148427
	H	-2.010273	-1.518358	-2.986182	H	-1.971590	-2.126016	-2.613546
	N	-0.353713	-0.603779	-1.975849	N	-0.324691	-1.021389	-1.799952
	H	0.232897	-0.730320	-2.805399	H	0.266982	-1.376361	-2.554972
	H	-2.091422	0.244093	-2.897185	H	-2.057513	-0.383846	-2.888419
	H	-3.540141	1.692438	-0.009850	H	-3.564370	1.609196	-0.362872
	H	-2.315161	1.693806	-1.305117	H	-2.335823	1.358814	-1.630082
	H	-1.916345	2.532779	1.611094	H	-1.954330	2.786430	1.050340
	H	-1.728247	3.483216	0.134553	H	-1.784267	3.416647	-0.590525
	H	0.445535	2.758051	0.778445	H	0.404340	2.885609	0.155151
	C	5.840991	0.329329	-0.009482	C	5.878070	0.289844	-0.019356
	H	5.726433	1.266463	-0.556607	H	5.796573	1.226487	-0.573144
	H	6.011016	-0.600443	-0.554584	H	6.033086	-0.647163	-0.556425
	H	5.907731	0.339992	1.079553	H	5.920365	0.304144	1.070834
	H	3.549580	-0.222877	0.052343	H	3.558280	-0.216372	0.013625
	Np	0.526458	-0.044456	-0.013414	Np	0.521715	-0.028950	0.001360
	O	2.621272	-0.491244	0.063604	O	2.626397	-0.469751	0.029824
TS2/radical	N	0.089712	-0.447303	1.995348	N	0.009468	-0.842411	1.834345
	C	1.454181	-0.694738	2.435008	C	1.335717	-1.307897	2.212670
	C	2.302592	-1.085199	1.219593	C	2.180803	-1.476312	0.945295
	N	2.024935	-0.209406	0.058559	N	2.001486	-0.336422	0.018084
	C	2.617769	1.137145	0.227620	C	2.683747	0.887275	0.498605
	C	1.946756	2.172496	-0.682125	C	2.093762	2.152399	-0.135202
	H	2.357427	2.091838	-1.713023	H	2.499359	2.288032	-1.161639
	H	2.259848	3.173720	-0.319591	H	2.473334	3.016021	0.448091
	N	0.506473	1.977585	-0.641288	N	0.641586	2.060377	-0.121932
	H	0.023098	2.776355	-1.061542	H	0.215422	2.973981	-0.299979
	H	3.714786	1.110151	0.059693	H	3.777653	0.825242	0.321336
	H	2.448702	1.433298	1.272824	H	2.524566	0.939161	1.585007
	C	2.421138	-0.835244	-1.223695	C	2.382007	-0.678632	-1.371375
	H	2.392550	-0.046523	-1.988804	H	2.450181	0.266200	-1.929037
	H	3.462413	-1.216709	-1.173385	H	3.382000	-1.159147	-1.401152
	C	1.447164	-1.943938	-1.635511	C	1.324962	-1.560281	-2.044189
	H	1.661412	-2.188326	-2.697126	H	1.546113	-1.565575	-3.130953
	N	0.085311	-1.476879	-1.421277	N	0.003446	-1.029386	-1.739829
	H	-0.580596	-2.140300	-1.830089	H	-0.698604	-1.441368	-2.361685

	H	1.672502	-2.874381	-1.068591	H	1.444057	-2.615458	-1.713435
	H	3.384697	-1.081219	1.468223	H	3.252687	-1.619764	1.195402
	H	2.032131	-2.108488	0.922790	H	1.838638	-2.377780	0.416820
	H	1.907065	0.183844	2.946362	H	1.843752	-0.615397	2.918871
	H	1.528908	-1.527571	3.164658	H	1.316581	-2.290506	2.726969
	H	-0.546532	-0.422929	2.797243	H	-0.644506	-0.940882	2.616622
	Np	-0.599499	0.141846	-0.043314	Np	-0.597364	0.234878	-0.012605
	O	-2.902209	0.574999	0.168233	O	-2.949069	0.504400	0.018448
	C	-4.102371	-0.791543	-0.043011	C	-3.859427	-1.017804	-0.003431
	H	-3.446536	1.338056	0.395645	H	-3.554230	1.209929	0.290217
	H	-3.445178	-1.636440	-0.265504	H	-3.105959	-1.756664	-0.290875
	H	-4.722577	-0.465498	-0.880368	H	-4.616376	-0.833191	-0.768277
	H	-4.601395	-0.852429	0.926271	H	-4.238567	-1.139302	1.013451
ECP	N	-0.161084	0.414711	1.970511	N	0.645801	-2.051663	-0.222291
	C	0.918350	1.307951	2.352629	C	2.090525	-2.160325	-0.348448
	C	2.100175	1.066043	1.408128	C	2.656248	-0.811100	-0.806237
	N	1.649106	0.915163	0.004224	N	2.040021	0.316886	-0.073110
	C	1.208547	2.210965	-0.565312	C	2.523507	0.411646	1.321861
	C	0.378680	2.032195	-1.840816	C	1.539148	1.184939	2.206085
	H	1.051777	1.792755	-2.689612	H	1.669475	2.279096	2.051835
	H	-0.052500	3.028077	-2.084116	H	1.831726	0.999458	3.260212
	N	-0.625799	0.996269	-1.631072	N	0.187773	0.736926	1.907366
	H	-0.983820	0.691857	-2.543486	H	-0.466081	1.067428	2.623234
	H	2.078343	2.874327	-0.752849	H	3.536487	0.864217	1.360744
	H	0.581672	2.697107	0.196140	H	2.601685	-0.614842	1.707021
	C	2.681175	0.258765	-0.835654	C	2.175121	1.597725	-0.799867
	H	2.363457	0.370197	-1.881637	H	1.890948	2.396727	-0.100234
	H	3.658690	0.772516	-0.723558	H	3.228526	1.773776	-1.102632
	C	2.803654	-1.238691	-0.528675	C	1.236194	1.658904	-2.009365
	H	3.408884	-1.682270	-1.347720	H	1.202624	2.716669	-2.343962
	N	1.470902	-1.811176	-0.421783	N	-0.066645	1.147181	-1.616674
	H	1.531480	-2.833836	-0.441219	H	-0.764526	1.343070	-2.339951
	H	3.411628	-1.383581	0.392922	H	1.677307	1.094511	-2.860412
	H	2.855374	1.875084	1.492120	H	3.762183	-0.788721	-0.710996
	H	2.586951	0.122344	1.691314	H	2.409407	-0.676752	-1.868951
	H	0.623764	2.381412	2.323152	H	2.578465	-2.472618	0.601371
	H	1.287399	1.135743	3.385564	H	2.401744	-2.914636	-1.100532
	H	-0.899466	0.423658	2.681001	H	0.218925	-2.979988	-0.162110
	Np	-0.428729	-0.666540	0.026807	Np	-0.574970	-0.221156	0.055737
	O	-2.865802	0.272691	-0.487017	O	-3.146474	-0.239058	-0.211711
	C	-3.838923	0.964749	0.289854	C	-4.204783	0.704434	0.062168
	H	-2.221543	0.915872	-0.933851	H	-3.508883	-1.032157	-0.642043
	H	-4.460023	0.216590	0.805867	H	-3.731454	1.580967	0.522249
	H	-4.497147	1.577750	-0.350258	H	-4.936453	0.272535	0.763242
	H	-3.368513	1.614494	1.049238	H	-4.702526	1.008619	-0.872173

Table S4. Cartesian coordinates (Å) of all intermediate and transition states for the reaction $N(CH_2CH_2NH)_3NpO$ (S=2,3) + CH_4 and for both [2+2] and radical mechanisms obtained at the B3LYP/RSC level of theory.

Species	S=2	S=3
ECR	N -0.152176 -0.740184 1.983921<br>C -1.385361 -0.321876 2.612088<br>C -2.130694 0.686932 1.728334<br>N -2.105451 0.310806 0.300630<br>C -2.875372 -0.915377 0.025020<br>C -2.357751 -1.659066 -1.212546<br>H -2.652176 -1.115143 -2.137821<br>H -2.876580 -2.636099 -1.274585<br>N -0.918774 -1.798723 -1.146859<br>H -0.619579 -2.586588 -1.730731<br>H -3.958008 -0.696840 -0.082070<br>H -2.758328 -1.588935 0.884204<br>C -2.512938 1.430840 -0.577838<br>H -2.623247 1.016559 -1.591622<br>H -3.503866 1.829290 -0.272343<br>C -1.461750 2.544703 -0.623991<br>H -1.740330 3.208543 -1.469016<br>N -0.143750 1.954256 -0.769937<br>H 0.533784 2.672069 -1.041734<br>H -1.556473 3.182982 0.284062<br>H -3.169989 0.819879 2.095944<br>H -1.630504 1.662584 1.809964<br>H -2.029133 -1.198502 2.839112<br>H -1.209886 0.155454 3.600062<br>H 0.361006 -1.366929 2.612281<br>C 5.685716 0.422977 0.653991<br>H 5.596840 0.934532 1.625119<br>H 6.160409 1.102735 -0.070274<br>H 6.313990 -0.473057 0.773711<br>H 4.688276 0.131224 0.290927<br>Np 0.571915 -0.130784 -0.226854<br>O 2.369457 -0.513590 -0.531546	N 0.264469 -1.277059 -1.632667<br>C -1.045910 -1.754113 -2.029384<br>C -1.977922 -1.827335 -0.810059<br>N -1.952194 -0.589752 -0.012762<br>C -2.815166 0.461852 -0.594872<br>C -2.480238 1.861275 -0.066110<br>H -2.934998 1.990534 0.943451<br>H -3.025026 2.578989 -0.715439<br>N -1.040959 2.058891 -0.074926<br>H -0.828723 3.051801 0.065605<br>H -3.889299 0.226279 -0.433556<br>H -2.636912 0.452244 -1.681625<br>C -2.237171 -0.826245 1.414878<br>H -2.307481 0.160773 1.894787<br>H -3.216336 -1.330488 1.561807<br>C -1.128144 -1.629730 2.108248<br>H -1.278866 -1.537221 3.205538<br>N 0.181986 -1.165157 1.703109<br>H 0.898763 -1.682644 2.223247<br>H -1.243256 -2.715858 1.904739<br>H -3.009060 -2.081687 -1.132856<br>H -1.621338 -2.649725 -0.175743<br>H -1.484395 -1.078522 -2.797291<br>H -1.014167 -2.754450 -2.502904<br>H 0.982036 -1.686943 -2.239328<br>C 3.778999 -1.785298 0.006681<br>H 4.224161 -2.156741 0.941875<br>H 4.337460 -2.191140 -0.850196<br>H 2.730601 -2.119246 -0.053750<br>H 3.831154 -0.684443 -0.011673<br>Np 0.593921 0.484711 -0.002697<br>O 2.688900 1.367429 0.006076
TS1/[2+2]	N -0.138843 -0.868088 1.859109<br>C -1.362081 -0.421802 2.490356<br>C -2.072288 0.620373 1.613496<br>N -2.018780 0.256377 0.186609<br>C -2.737682 -0.992442 -0.112528<br>C -2.075633 -1.758587 -1.266101<br>H -2.303395 -1.256131 -2.231834<br>H -2.532560 -2.763825 -1.336363<br>N -0.644395 -1.797445 -1.063330	N/A

	H	-0.243097	-2.658362	-1.449311	
	H	-3.807446	-0.803907	-0.335619	
	H	-2.697330	-1.637388	0.774031	
	C	-2.433612	1.355820	-0.708117	
	H	-2.550603	0.919914	-1.712154	
	H	-3.421387	1.765252	-0.406715	
	C	-1.382634	2.464628	-0.789833	
	H	-1.667439	3.118587	-1.639415	
	N	-0.068748	1.864340	-0.950980	
	H	0.605666	2.578024	-1.246848	
	H	-1.451445	3.114342	0.111270	
	H	-3.114995	0.766533	1.967656	
	H	-1.559552	1.588739	1.718923	
	H	-2.035619	-1.274967	2.712380	
	H	-1.163172	0.041367	3.480751	
	H	0.306116	-1.596876	2.425798	
	C	2.817281	1.087932	1.086602	
	H	2.341785	1.099336	2.086588	
	H	2.707425	2.101080	0.654404	
	H	3.898259	0.935333	1.257507	
	H	2.879377	-0.152318	0.200671	
	Np	0.765954	-0.086935	-0.182925	
	O	2.518871	-1.015197	-0.515353	
IC/[2+2]	N	0.152524	0.683402	1.896759	N/A
	C	-1.152971	1.185112	2.304183	
	C	-1.993810	1.482588	1.057759	
	N	-1.898735	0.391711	0.060385	
	C	-2.647384	-0.815845	0.480677	
	C	-2.132779	-2.072022	-0.230490	
	H	-2.552891	-2.125966	-1.258713	
	H	-2.553483	-2.946691	0.304857	
	N	-0.677235	-2.060745	-0.225132	
	H	-0.304519	-3.000227	-0.379463	
	H	-3.736737	-0.679697	0.318102	
	H	-2.483884	-0.938446	1.560505	
	C	-2.287390	0.835580	-1.299155	
	H	-2.419754	-0.071121	-1.906533	
	H	-3.260736	1.368228	-1.275860	
	C	-1.202545	1.697816	-1.952070	
	H	-1.456042	1.778628	-3.028950	
	N	0.100550	1.088024	-1.722027	
	H	0.794189	1.490339	-2.358332	
	H	-1.257984	2.737124	-1.559205	
	H	-3.052006	1.678529	1.329291	
	H	-1.600794	2.391563	0.579235	
	H	-1.694208	0.472430	2.964046	

	H	-1.088309	2.128635	2.884574				
	H	0.786762	0.654053	2.699459				
	C	2.822157	2.217424	0.072383				
	H	2.102990	2.227067	0.895670				
	H	2.669137	2.878566	-0.781668				
	H	3.749480	1.649895	0.163069				
	H	3.415334	-1.562363	-0.231638				
	Np	0.658112	-0.298425	-0.050209				
	O	2.570961	-1.284708	0.135173				
TS1/radical	N	0.055803	-1.051222	1.762928	N	-0.189999	-1.405563	-1.649408
	C	1.413433	-1.451904	2.107177	C	-1.570551	-1.484882	-2.079740
	C	2.276255	-1.460058	0.839832	C	-2.513586	-1.274268	-0.886540
	N	2.040605	-0.258485	0.003882	N	-2.140330	-0.098523	-0.086296
	C	2.666903	0.955862	0.579296	C	-2.615790	1.163065	-0.693466
	C	2.013768	2.235276	0.045757	C	-1.884912	2.390794	-0.137645
	H	2.407740	2.468707	-0.967400	H	-2.327715	2.666381	0.848058
	H	2.353650	3.069328	0.692371	H	-2.142281	3.237628	-0.808063
	N	0.566303	2.073671	0.054899	N	-0.458689	2.125456	-0.073706
	H	0.099014	2.978385	-0.043652	H	0.056880	3.008486	-0.030678
	H	3.761225	0.957694	0.396575	H	-3.716318	1.268762	-0.575289
	H	2.510650	0.912852	1.666731	H	-2.404711	1.095273	-1.771925
	C	2.431779	-0.475278	-1.409575	C	-2.537135	-0.218659	1.330264
	H	2.431896	0.509772	-1.897768	H	-2.309641	0.745819	1.806829
	H	3.462106	-0.881927	-1.473828	H	-3.630785	-0.384702	1.435936
	C	1.436184	-1.380392	-2.144197	C	-1.772801	-1.322138	2.074486
	H	1.657412	-1.293808	-3.227508	H	-1.956637	-1.178844	3.161941
	N	0.078256	-0.968930	-1.815384	N	-0.361483	-1.287980	1.754291
	H	-0.590066	-1.407192	-2.455359	H	0.119197	-2.029501	2.275822
	H	1.630101	-2.445017	-1.888625	H	-2.207975	-2.319641	1.850625
	H	3.352653	-1.557999	1.090380	H	-3.564953	-1.212487	-1.237785
	H	1.994755	-2.333343	0.234515	H	-2.433981	-2.162721	-0.245877
	H	1.868194	-0.784179	2.871082	H	-1.772174	-0.719351	-2.862116
	H	1.463681	-2.471277	2.540467	H	-1.816731	-2.456062	-2.552886
	H	-0.594330	-1.309861	2.510048	H	0.398689	-1.948900	-2.289856
	C	-4.760867	-0.675700	0.008970	C	5.014453	-0.550869	-0.069400
	H	-4.431468	-1.723060	0.042685	H	5.589474	-0.082162	0.740440
	H	-5.302051	-0.440183	-0.916989	H	5.476678	-0.389296	-1.052499
	H	-5.324110	-0.388805	0.906891	H	4.819450	-1.614410	0.124890
	H	-3.778373	0.020898	0.002005	H	3.951079	0.019339	-0.101351
	Np	-0.558283	0.162573	-0.002184	Np	0.615110	0.129924	0.058695
	O	-2.637982	0.743456	-0.010919	O	2.750331	0.602681	-0.127102
IC/radical	N	-0.230360	-2.083542	-0.012964	N	-0.402502	-1.336684	-1.698684
	C	-1.624839	-2.506837	-0.027984	C	-1.759847	-1.197867	-2.181445
	C	-2.500231	-1.386067	0.544202	C	-2.707758	-0.892192	-1.013696
	N	-2.121556	-0.057812	0.005477	N	-2.188297	0.177496	-0.149245
	C	-2.554458	0.121418	-1.400469	C	-2.425600	1.520378	-0.718644

	C	-1.740098	1.208949	-2.110058	C	-1.539852	2.596358	-0.077673
	H	-2.120367	2.214313	-1.824671	H	-1.986765	2.905110	0.896576
	H	-1.948001	1.114546	-3.195527	H	-1.627014	3.495766	-0.723745
	N	-0.330047	1.038121	-1.789971	N	-0.178091	2.107408	0.044405
	H	0.253355	1.586921	-2.426109	H	0.459156	2.898993	0.169072
	H	-3.641762	0.336887	-1.453460	H	-3.499864	1.798592	-0.643712
	H	-2.379113	-0.834255	-1.914946	H	-2.175609	1.460315	-1.789186
	C	-2.577998	1.049469	0.877982	C	-2.661838	0.072684	1.244197
	H	-2.462178	1.979626	0.303553	H	-2.304075	0.969856	1.769653
	H	-3.654309	0.938413	1.124693	H	-3.771869	0.082439	1.301339
	C	-1.727992	1.156771	2.149349	C	-2.118972	-1.165137	1.971369
	H	-1.960705	2.136176	2.614278	H	-2.334056	-1.035028	3.055089
	N	-0.320616	1.021642	1.800110	N	-0.706516	-1.348920	1.713978
	H	0.273373	1.370108	2.556383	H	-0.377478	-2.170914	2.232358
	H	-2.056488	0.394629	2.889805	H	-2.695681	-2.070862	1.684315
	H	-3.576373	-1.590622	0.365899	H	-3.723125	-0.654412	-1.395394
	H	-2.345218	-1.346957	1.631940	H	-2.789955	-1.806625	-0.410940
	H	-1.974224	-2.777337	-1.048483	H	-1.819414	-0.387407	-2.942437
	H	-1.806668	-3.408228	0.592363	H	-2.124715	-2.107494	-2.699559
	H	0.384681	-2.890225	-0.154174	H	0.142254	-1.898423	-2.361325
	C	5.946239	-0.254914	0.021520	C	6.153304	0.128979	-0.121861
	H	5.904580	-1.147600	0.647606	H	6.106125	0.638095	-1.085484
	H	6.057264	0.727828	0.482236	H	6.277593	-0.954130	-0.083202
	H	5.977850	-0.352376	-1.064748	H	6.130807	0.708502	0.802118
	H	3.560322	0.169727	-0.014557	H	3.687225	-0.076520	-0.147629
	Np	0.517815	0.023437	-0.002185	Np	0.573065	-0.045337	0.105281
	O	2.631444	0.429653	-0.031837	O	2.727828	-0.144224	-0.175714
TS2/radical	N	0.174407	-0.357456	2.040898	N	0.243985	0.978893	1.832169
	C	1.559389	-0.560061	2.437424	C	1.473869	0.553905	2.466697
	C	2.369684	-0.988248	1.208538	C	2.117804	-0.590247	1.670813
	N	2.033701	-0.171229	0.019985	N	2.063477	-0.350825	0.215881
	C	2.596539	1.195265	0.112729	C	2.887658	0.795677	-0.197832
	C	1.872931	2.174129	-0.818566	C	2.294039	1.521887	-1.414141
	H	2.252073	2.055124	-1.857992	H	2.441692	0.905159	-2.328697
	H	2.177716	3.196832	-0.512017	H	2.867614	2.452458	-1.586402
	N	0.439046	1.953779	-0.721821	N	0.879871	1.754719	-1.208558
	H	-0.068983	2.710331	-1.187935	H	0.590953	2.627248	-1.663319
	H	3.688318	1.187556	-0.088329	H	3.933813	0.491046	-0.406736
	H	2.452220	1.532121	1.149282	H	2.916230	1.515814	0.629944
	C	2.408789	-0.843440	-1.244939	C	2.367367	-1.563168	-0.573506
	H	2.337737	-0.090364	-2.042527	H	2.440018	-1.245273	-1.625779
	H	3.460878	-1.196174	-1.208637	H	3.353633	-1.987580	-0.285569
	C	1.453458	-1.993637	-1.580451	C	1.267460	-2.625197	-0.468126
	H	1.648410	-2.279780	-2.635352	H	1.479554	-3.377074	-1.257110
	N	0.085268	-1.553475	-1.353235	N	-0.032981	-1.993675	-0.611790
	H	-0.571183	-2.235934	-1.744948	H	-0.749131	-2.708275	-0.780269

	H	1.719234	-2.891796	-0.979420	H	1.382937	-3.179534	0.492050
	H	3.459993	-0.953425	1.416211	H	3.157995	-0.761620	2.021632
	H	2.109542	-2.028841	0.967557	H	1.553464	-1.514235	1.864487
	H	2.014453	0.348220	2.892365	H	2.184183	1.399624	2.583263
	H	1.680229	-1.358463	3.198968	H	1.303902	0.188650	3.503008
	H	-0.427882	-0.311284	2.867946	H	-0.181797	1.741383	2.371279
	Np	-0.601881	0.109338	-0.013917	Np	-0.650551	0.153888	-0.207936
	O	-2.874729	0.629599	0.015123	O	-3.009727	0.494169	-0.076188
	C	-4.204245	-0.750228	-0.003449	C	-4.070914	-0.730389	0.695584
	H	-3.360732	1.360348	0.415718	H	-3.551980	0.992923	-0.711272
	H	-3.622600	-1.583714	-0.403389	H	-3.383477	-1.228411	1.383994
	H	-4.942693	-0.326990	-0.686033	H	-4.439383	-1.362663	-0.115320
	H	-4.500214	-0.842975	1.043357	H	-4.825359	-0.113187	1.187543
ECP	N	-0.191471	0.432694	1.967488	N	0.350108	-0.763190	1.932913
	C	0.878811	1.338180	2.346400	C	1.075066	0.323046	2.565595
	C	2.069483	1.100048	1.411791	C	2.175017	0.831552	1.619375
	N	1.631663	0.936497	0.005655	N	1.668789	1.028426	0.247163
	C	1.177078	2.222740	-0.573550	C	1.128894	2.389282	0.053535
	C	0.360356	2.028357	-1.855339	C	0.282060	2.518933	-1.214779
	H	1.044365	1.793393	-2.696859	H	0.952735	2.540558	-2.100734
	H	-0.078614	3.019025	-2.106789	H	-0.177719	3.530822	-1.183135
	N	-0.635072	0.982815	-1.650915	N	-0.692504	1.438325	-1.275907
	H	-0.986584	0.678583	-2.565585	H	-1.174537	1.473790	-2.184156
	H	2.038925	2.897711	-0.757076	H	1.942829	3.145796	0.052693
	H	0.537341	2.703531	0.180588	H	0.488561	2.600116	0.925945
	C	2.677934	0.290451	-0.824426	C	2.659425	0.650884	-0.776530
	H	2.360104	0.384337	-1.872230	H	2.230577	0.906112	-1.756320
	H	3.644796	0.825165	-0.716604	H	3.604443	1.225171	-0.671994
	C	2.833050	-1.200929	-0.502354	C	2.950486	-0.855248	-0.750049
	H	3.457190	-1.634960	-1.312868	H	3.463909	-1.125799	-1.698194
	N	1.515511	-1.807858	-0.399176	N	1.718986	-1.596755	-0.600310
	H	1.609965	-2.827858	-0.388766	H	1.917681	-2.602472	-0.533039
	H	3.439373	-1.320768	0.424390	H	3.678697	-1.112160	0.045672
	H	2.816999	1.916252	1.497008	H	2.630812	1.761349	2.015029
	H	2.562481	0.162550	1.704819	H	2.965517	0.070721	1.593497
	H	0.575842	2.409181	2.306185	H	0.382260	1.159458	2.804159
	H	1.244695	1.179401	3.382912	H	1.545892	0.036618	3.524326
	H	-0.934625	0.448919	2.672558	H	0.200137	-1.543435	2.581552
	Np	-0.422368	-0.682729	0.025560	Np	-0.516310	-0.676860	-0.251543
	O	-2.868860	0.263733	-0.493447	O	-2.811431	0.547200	0.003271
	C	-3.813593	0.981765	0.294114	C	-3.783283	1.000568	0.939391
	H	-2.214839	0.893899	-0.950136	H	-2.236619	1.284748	-0.366389
	H	-4.455449	0.251575	0.810822	H	-4.303833	0.117139	1.337632
	H	-4.456885	1.619746	-0.336955	H	-4.526186	1.655513	0.452516
	H	-3.317061	1.611538	1.053902	H	-3.320478	1.544583	1.782061
